# High‐Throughput Screening Using the Self‐Controlled Tree‐Based Scan Statistic to Identify Medications Associated With Hospitalization for Severe Acute Liver Injury

**DOI:** 10.1002/pds.70275

**Published:** 2025-12-04

**Authors:** Vincent Lo Re, Craig W. Newcomb, Dean M. Carbonari, Charles E. Leonard, Christopher T. Rentsch, Judith C. Maro

**Affiliations:** ^1^ Division of Infectious Diseases, Department of Medicine, Perelman School of Medicine University of Pennsylvania Philadelphia Pennsylvania USA; ^2^ Center for Clinical Epidemiology and Biostatistics, Center for Real‐World Effectiveness and Safety of Therapeutics, Department of Biostatistics, Epidemiology, and Informatics, Perelman School of Medicine University of Pennsylvania Philadelphia Pennsylvania USA; ^3^ Leonard Davis Institute of Health Economics University of Pennsylvania Philadelphia Pennsylvania USA; ^4^ Department of Non‐communicable Disease Epidemiology London School of Hygiene & Tropical Medicine London UK; ^5^ VA Connecticut Healthcare System, US Department of Veterans Affairs West Haven Connecticut USA; ^6^ Department of Medicine, Yale School of Medicine New Haven Connecticut USA; ^7^ Department of Population Medicine Harvard Medical School and Harvard Pilgrim Healthcare Institute Boston Massachusetts USA

**Keywords:** acute liver injury, drug‐induced liver injury, hepatotoxicity, high‐throughput screening, tree‐based scan statistics

## Abstract

**Background:**

Medications associated with acute liver injury (ALI) are primarily identified by case reports. High‐throughput screening of real‐world data could be leveraged to detect hepatotoxicity signals.

**Objective:**

To apply tree‐based scan statistics in real‐world data to identify drugs associated with hospitalization for severe ALI among patients without liver/biliary disease and with chronic liver disease (CLD).

**Methods:**

We implemented a self‐controlled case‐crossover design in Veterans Health Administration data (2000–2023) among patients hospitalized for laboratory‐confirmed severe ALI. We identified all newly dispensed drugs within 365 days prior to their hospitalization and used conditional Bernoulli tree‐based scan statistics to identify potential associations (*p* < 0.3). We performed analyses separately in patients without liver/biliary disease and with CLD.

**Results:**

Among 12 860 patients without liver/biliary disease and 17 512 with CLD hospitalized for severe ALI, we evaluated associations with 450 and 543 drugs, respectively. Drugs associated with severe ALI among patients without liver/biliary disease included: acid‐suppressives (ranitidine [*p* < 0.001], omeprazole [*p* = 0.004]), antiemetics (ondansetron [*p* < 0.001], promethazine [*p* = 0.06]), antibiotics (amoxicillin/clavulanate [*p* = 0.008], ciprofloxacin [*p* = 0.02], mupirocin [*p* = 0.032], ethambutol [*p* = 0.275]), anticoagulants (heparin [*p* = 0.015]), and chemotherapy (pazopanib [*p* = 0.275]). Drugs associated with severe ALI among CLD patients were: diuretics (spironolactone, furosemide [both *p* < 0.001]), antiemetics (ondansetron, metoclopramide, promethazine [all *p* < 0.001]), appetite stimulants (*p* < 0.001), analgesics (morphine, oxycodone, fentanyl [all *p* < 0.001]), chemotherapy (sorafenib [*p* < 0.001]), antibiotics (ciprofloxacin [*p* = 0.011], metronidazole [*p* = 0.020]), antipsychotics (prochlorperazine [*p* = 0.105]), vitamins (*p* = 0.134), acid‐suppressives (omeprazole [*p* = 0.164]), and gastrointestinal/liver disease treatments (lactulose, senna, docusate, silicones, antiflatulents [all *p* < 0.001]; sucralfate [*p* = 0.005], albumin [*p* = 0.228]).

**Conclusions:**

High‐throughput screening using tree‐based scan statistics detected potentially hepatotoxic drugs for investigation in future pharmacoepidemiology studies.

## Key Points

- Drugs associated with acute liver injury (ALI) are primarily identified by case reports. High‐throughput screening of real‐world data could detect hepatotoxicity signals.
- We implemented the tree‐based scan statistic in US Veterans Health Administration data to identify drugs possibly associated with hospitalization for severe ALI among patients without liver/biliary disease and with chronic liver disease (CLD).
- Among 12 860 patients without liver/biliary disease hospitalized for severe ALI, we evaluated associations with 450 newly dispensed drugs within 365 days prior to hospitalization. Drugs associated with severe ALI were: antiemetics (ondansetron, promethazine), acid‐suppressives (ranitidine, omeprazole), antibiotics (amoxicillin/clavulanate, ciprofloxacin, mupirocin, ethambutol), anticoagulants (heparin), and chemotherapy (pazopanib).
- Among 17 512 patients with CLD hospitalized for severe ALI, we examined associations with 543 newly dispensed drugs within 365 days prior to hospitalization. Drugs associated with severe ALI were: diuretics (spironolactone, furosemide), antiemetics (ondansetron, metoclopramide, promethazine), appetite stimulants, analgesics (morphine, oxycodone, fentanyl), chemotherapy (sorafenib), antibiotics (ciprofloxacin, metronidazole), antipsychotics (prochlorperazine), vitamins, acid‐suppressives (omeprazole), and gastrointestinal/liver disease treatments (lactulose, senna, docusate, silicones, antiflatulents, sucralfate, albumin).
- High‐throughput screening using tree‐based scan statistics can detect potentially hepatotoxic drugs for investigation in future pharmacoepidemiology studies.

## 1 Introduction

Drug‐induced acute liver injury (ALI) is the most common cause of acute liver failure in the United States (US) and Europe [1, 2, 3], and is a major cause of acute liver failure in the Asia‐Pacific region [4], Africa [5], and Latin America [6]. Despite its clinical importance, no systematic approach has been taken to evaluate medications associated with severe ALI. Hepatotoxic drugs are primarily identified by post‐marketing spontaneous adverse event reports [7]. However, case reports of hepatotoxicity are limited by underreporting of events, misclassification of a drug‐induced etiology for ALI, and lack of information on the number exposed [8, 9]. We previously performed a series of new‐user cohort studies evaluating rates of severe ALI following initiation of 194 medications with at least four published reports of hepatotoxicity, but these analyses excluded medications with ≤ 3 published reports of drug‐induced ALI or more recently approved drugs [10]. As a result, there remain major knowledge gaps regarding medications associated with severe ALI.

To overcome these limitations, high‐throughput screening of real‐world data could be used to comprehensively evaluate associations between medication exposure and severe ALI. Application of high‐throughput screening methods, particularly tree‐based scan statistics [11, 12, 13], within large electronic health record data could detect hepatotoxicity signals from drug‐specific effects. Signals identified with these methods could then be further investigated using formal pharmacoepidemiologic methods, such as emulated trials [14, 15] or active comparator, new user cohort studies [16]. From a methodologic standpoint, high‐throughput screening would allow evaluation of thousands of medications in a rapid, efficient manner to detect possible associations with severe ALI. The large pool of existing medications could then be narrowed to a more limited number of potentially hepatotoxic drugs that could be more rigorously examined in causal inference‐based studies. From a clinical standpoint, this approach could enable differentiation of medications with little likelihood for severe ALI from those with increased potential for hepatotoxicity. Such information could inform clinicians' decision‐making regarding the selection of medications within a drug class as well as the need for monitoring for severe ALI following the initiation of drugs known to be associated with severe ALI. However, to date, these novel signal detection approaches have not been applied within real‐world data to detect hepatotoxicity safety signals.

Our objective was to perform high‐throughput screening using the tree‐based scan statistic in real‐world data to identify drugs potentially associated with hospitalization for severe ALI. The tree‐based scan statistic method allows a wide variety of drug exposures to be simultaneously evaluated to identify potential hepatotoxic effects, adjusting for multiplicity [11, 17]. Since chronic liver disease (CLD) might affect drug pharmacokinetics and hepatic drug metabolism [18], we performed high‐throughput screening separately among patients without pre‐existing liver disease and in those with diagnosed CLD.

## 2 Methods

### 2.1 Data Source and Study Design

We used electronic health record data from the US Veterans Health Administration (VA) between April 1, 2000 and April 30, 2023. The VA comprises > 1300 points of care nationwide, including hospitals, medical centers, and community outpatient clinics. VA electronic health record data are accessible from the Corporate Data Warehouse within the VA's Informatics and Computing Infrastructure [19]. Available data included: demographics; visit dates; outpatient and hospital diagnoses (recorded by International Classification of Diseases, Ninth Revision [ICD‐9] or Tenth Revision [ICD‐10] Clinical Modification diagnostic codes); laboratory orders with results; and dispensed medications.

**FIGURE 1:**
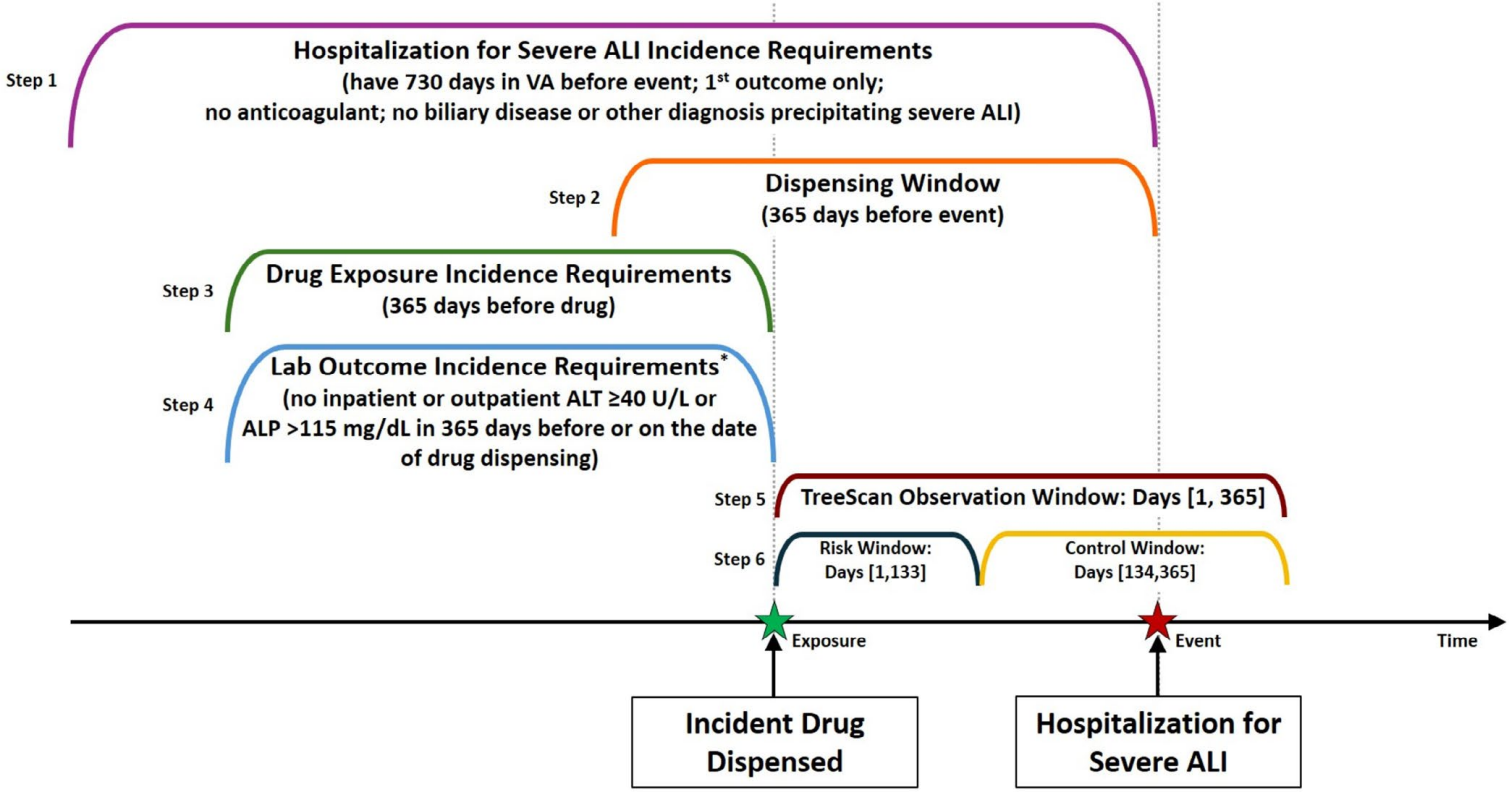
Design diagram for the primary analysis. ALI, acute liver injury; ALP, alkaline phosphatase; ALT, alanine aminotransferase; VA, Veterans Health Administration. * Only applicable to persons without chronic liver disease.

We implemented the tree‐based scan statistic within a self‐controlled case‐crossover design among patients who had a hospitalization for severe ALI in the VA system [20]. The self‐controlled case‐crossover design was utilized because it is outcome‐anchored (as opposed to exposure‐anchored) and minimizes bias by inherently adjusting for time‐invariant confounding [21, 22]. Incident hospitalizations for severe ALI were identified, and incident drug exposures in a period preceding the outcome were then scanned over relevant time periods (Figure 1). By design, the observation window always contains the incident outcome.

This study was approved by the Institutional Review Boards of the University of Pennsylvania, VA Connecticut Healthcare System, and Yale University.

### 2.2 Study Patients

Eligible patients had: (1) a hospitalization for severe ALI and (2) ≥ 730 days since first visit in the VA system prior to their hospitalization. Hospitalization for severe ALI was defined by meeting either of the following definitions within the first 2 days of hospital admission: (1) alanine aminotransferase (ALT) > 120 U/L (3 times upper limit of normal [ULN], 40 U/L) + total bilirubin > 2.0 mg/dL (2 times ULN, 1.0 mg/dL) (definition 1) or (2) international normalized ratio ≥ 1.5 + total bilirubin > 2.0 mg/dL (definition 2). Definition 1 represents Hy's Law biochemical criteria [23], which indicate hepatocellular injury severe enough to interfere with bilirubin excretion and predisposes to high risk of death [24, 25]. Definition 2 indicates hepatic dysfunction that might occur in an advanced stage of acute liver failure [26]. We classified severe ALI that occurred within the first 2 days of admission to avoid capturing outcomes that developed as a consequence of events that occurred during the hospitalization. For patients with more than one qualifying hospitalization in the study period, we limited inclusion to only the first hospitalization. We defined the date of admission as the index date.

Patients were excluded if, during the 730 days prior to the index date, they had: (1) a dispensing for warfarin or a direct oral anticoagulant (apixaban, dabigatran, edoxaban, rivaroxaban), since these would prevent ascertainment of ALI‐induced coagulopathy or (2) acute liver disease, biliary disease, or another condition that might precipitate findings consistent with severe ALI (Table S1). Patients who had a hospital discharge diagnosis (principal or contributory) of acute liver disease, biliary disease, or another condition that might precipitate findings consistent with severe ALI (Table S1) were also excluded to increase the likelihood that severe ALI events were medication related.

Eligible patients were then classified by CLD status. We defined a patient as having CLD if, during their hospitalization for severe ALI or within 730 days prior, they had: (1) ≥ 1 hospital or ≥ 2 outpatient ICD‐9/−10 diagnoses of CLD (Table S2) or (2) a positive laboratory test for hepatitis B virus infection (hepatitis B surface antigen, hepatitis B e antigen, or hepatitis B DNA) or hepatitis C virus infection (hepatitis C RNA or genotype).

### 2.3 Ascertainment of Medication Exposure

All drugs newly dispensed in the outpatient setting within 365 days prior to a hospitalization for severe ALI were identified by mapping National Drug Codes and Healthcare Common Procedure Coding System codes to the Anatomical Therapeutic Chemical (ATC) Classification System [27]. Drugs were considered incident if there was no previous dispensing of that medication in the preceding 365 days relative to the identified dispensing (i.e., 365‐day washout period). To further minimize inclusion of patients with undiagnosed acute liver or biliary disease, among patients without CLD, we additionally excluded from evaluation any dispensed drugs for patients who had ALT ≥ 40 U/L or alkaline phosphatase ≥ 115 mg/dL (ULNs of these laboratory tests) recorded in the inpatient or outpatient setting within the 365 days prior to an incident fill (Figure 1).

### 2.4 Statistical Analysis

We implemented the conditional Bernoulli tree‐based scan statistic for fixed risk (i.e., focal) window analyses separately among patients without liver/biliary disease and in those with diagnosed CLD using TreeScan software version 2.2. Details are presented in the Appendix S1. Tree‐based scan statistics are used in pharmacovigilance to identify new and unexpected safety signals for drugs using real‐world healthcare data. The tree structure is advantageous because it permits screening across thousands of drug exposures (referred to as “nodes”) simultaneously to identify potential individual drug hepatotoxic effects. Prior studies have used simulated data to evaluate the ability of tree‐based scan statistics to detect an array of hypothetical risk estimates and have shown that the tree‐based scan statistic methodology works under controlled conditions [28, 29, 30, 31]. In addition, there have been empirical studies with known positives that have been detected [13, 32].

We defined the fixed risk window as the 1–133 days after each incident drug fill, since we previously observed that a sizeable majority of hospitalizations for severe ALI occurred within this period following drug initiation regardless of CLD status [33]. Details of the conditional Bernoulli tree‐based scan statistic have been described previously and are additionally presented in the Appendix S1 [11, 29]. Briefly, the Bernoulli tree‐based scan statistic compares the proportion of ALI events in the risk window (1–133 days after incident dispensing) relative to the entire 365‐day observation window following incident dispensing for each drug being assessed. The assumption is that the proportion of ALI events that occur after a specific drug in this window is the same as the proportion of ALI events that occur after any drug in this window. Log‐likelihood ratios (LLR) are calculated for every drug assessed, and the maximum LLR is the test statistic *T*. A Monte Carlo‐based *p*‐value for *T* is obtained by generating random datasets under the composite null hypothesis that every ALI event following a specific drug exposure occurs with the same expected probability during the risk window of 1–133 days as the overall occurrence of ALI events following all drug exposures during the risk window of 1–133 days. This method formally adjusts for the multiple drugs being assessed by using the maximum of the LLR across all the drugs assessed in the tree and is explained in Appendix S2.

Although a key aspect of the tree‐based scan statistic is the use of a hierarchical tree to evaluate drugs and classes, in this study, we evaluated medications solely at the ATC Classification System level 5 (i.e., drug level) without a hierarchical structure because our primary goal was to identify individual medications potentially associated with severe ALI. To ensure sufficient precision to estimate associations, in the primary analysis, we only evaluated medications with a minimum of five cases of severe ALI. We classified drugs with *p* < 0.3 as “alerts,” indicating a potential association with severe ALI. Since this is a hypothesis‐generating study, we selected a higher *p*‐value cut‐off of 0.3 for alerts to seek to identify potentially unsuspected hepatotoxic drugs [34].

To enhance our ability to identify alerts, we performed several sensitivity analyses, stratified by CLD status. First, since drugs known to be potential treatments for ALI symptoms may be detected due to protopathic bias and potentially mask important associations between drugs and severe ALI, we repeated the analysis after pruning such medications in ATC branch group A (i.e., drugs to treat alimentary tract/metabolic conditions, such as antiemetic and antispasmodic drugs) from the tree of drugs to be examined, with the exception of medications in branch group A02 (i.e., acid‐suppressive drugs). Second, since associations between medications and severe ALI may differ in varying risk windows after drug initiation, we repeated the analysis evaluating risk windows of 1–84 and 1–28 days. Third, to identify other potential drugs of interest, we repeated the primary analysis, increasing the minimum number of required cases of severe ALI for each medication from five to 10. Increasing the minimum number of required severe ALI cases reduces the scanning of multiple medications and might unmask additional alerts.

## 3 Results

### 3.1 Patients Without Liver/Biliary Disease

**FIGURE 2:**
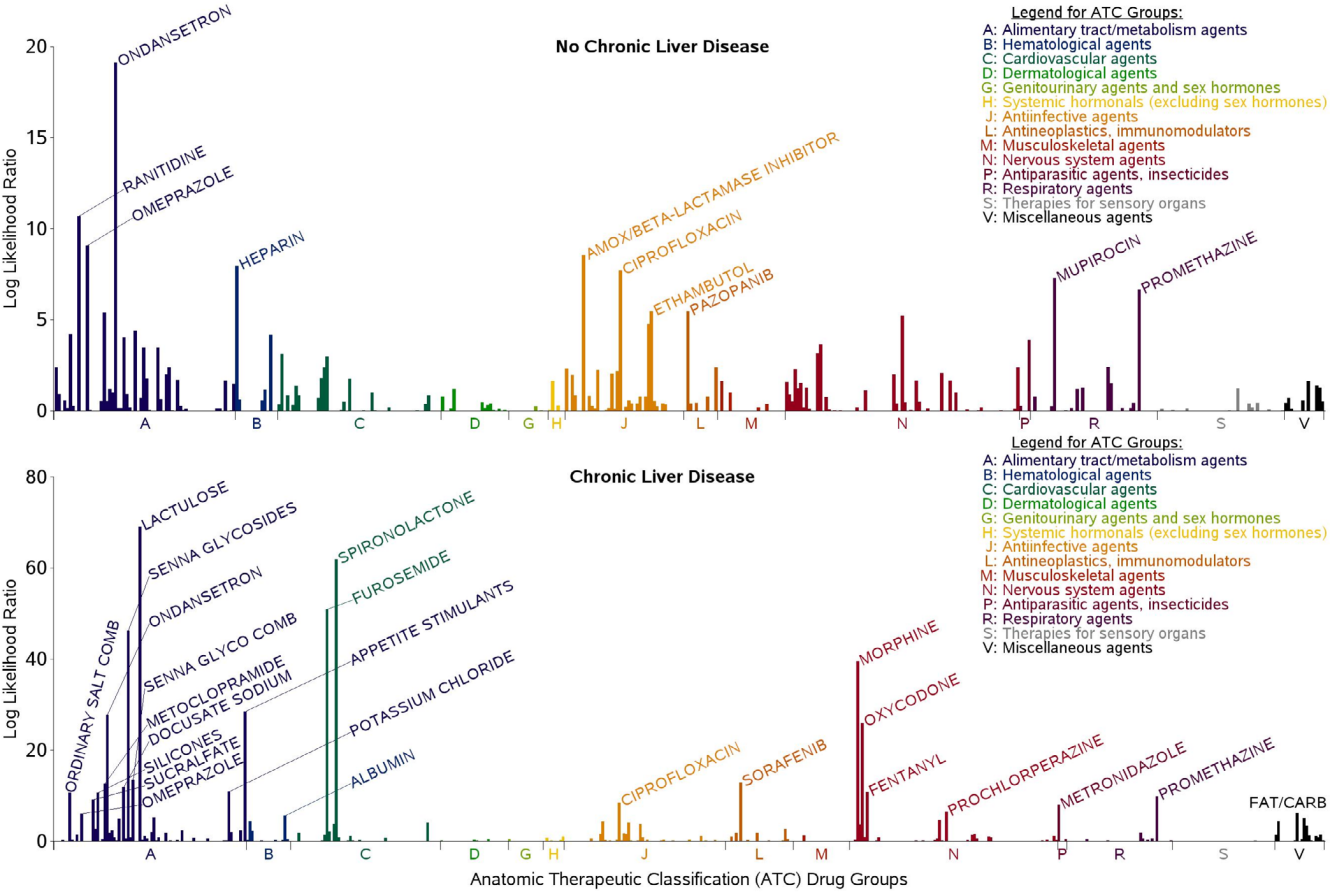
Log likelihood ratio for drugs in the Anatomic Therapeutic Classification drug groups by chronic liver disease status. Peaks with labels qualify as alerts. ATC, Anatomic Therapeutic Classification; carb, carbohydrates; CLD, chronic liver disease; comb, combination; glyco, glycoside.

Between April 1, 2000 and April 30, 2023, we identified 12 860 patients in the VA system without liver/biliary disease who had a hospitalization for severe ALI. Among these 12 860 patients, we identified 44 872 new dispensings of 450 unique drugs within 365 days prior to a hospitalization for severe ALI in the primary analysis. Of these, 18 091 new dispensings (40.3%) were within the first 133 days. Among the 450 unique drugs screened for possible association with severe ALI, those identified as alerts were from the following classes (Table 1; Figure 2): acid‐suppressives (ranitidine [*p* < 0.001], omeprazole [*p* = 0.004]), antiemetics (ondansetron [*p* < 0.001], promethazine [*p* = 0.06]), antibiotics (amoxicillin/clavulanate [*p* = 0.008], ciprofloxacin [*p* = 0.02], mupirocin [*p* = 0.032], ethambutol [*p* = 0.275]), anticoagulants (heparin [*p* = 0.015]), and chemotherapy (pazopanib [*p* = 0.275]).

**TABLE 1:** Drugs with *p* < 0.3 in days 1–133 among people without chronic liver disease.

Node	Drug description	Number of severe ALI cases	Observed cases in window	Expected cases in window	Observed/Expected	Observed‐expected	Test statistic	*p*
A04AA01	Ondansetron	286	167	115.20	1.45	52.12	19.13	0.001
A02BA02	Ranitidine	727	354	292.85	1.21	62.15	10.68	0.001
A02BC01	Omeprazole	1190	551	479.35	1.15	73.58	9.09	0.004
J01CR02	Amoxicillin/beta‐lactamase inhibitor	489	242	196.98	1.23	45.51	8.55	0.008
B01AB01	Heparin	32	24	12.89	1.86	11.12	7.96	0.015
J01MA02	Ciprofloxacin	698	332	281.16	1.18	51.63	7.70	0.022
R01AX06	Mupirocin	21	17	8.46	2.01	8.54	7.30	0.032
R06AD02	Promethazine	172	93	69.28	1.34	23.81	6.66	0.064
J04AK02	Ethambutol	6	6	2.42	2.48	3.58	5.46	0.275
L01EX03	Pazopanib	6	6	2.42	2.48	3.58	5.46	0.275

Sensitivity analyses conducted after pruning medications in ATC branch group A (with the exception of branch group A02, acid‐suppressive drugs; Table S3) additionally identified an alert for prochlorperazine (*p* = 0.125). The sensitivity analysis evaluating a shorter risk window of 1–84 days (Table S4) additionally identified alerts for hydrocodone/acetaminophen (*p* = 0.005), drugs used to treat gastrointestinal/liver diseases (dicyclomine [*p* = 0.004], antiflatulents [*p* = 0.130], docusate [*p* = 0.161]), prochlorperazine (*p* = 0.061), lidocaine (*p* = 0.240), and appetite stimulants (*p* = 0.266). The sensitivity analysis evaluating a risk window of 1–28 days (Table S5) additionally identified alerts for hydrocodone/acetaminophen (*p* < 0.001), gatifloxacin (*p* = 0.021), gastrointestinal/liver disease treatments (bismuth [*p* = 0.022], docusate [*p* = 0.026], antiflatulents [*p* = 0.061], dicyclomine [*p* = 0.066], lactulose [*p* = 0.294]), hyoscyamine (*p* = 0.152), and appetite stimulants (*p* = 0.276). When we repeated the primary analysis increasing the minimum number of required severe ALI cases for each medication from five to 10 (Table S6), the number of unique drugs evaluated decreased from 450 to 352, and we additionally identified alerts for dicyclomine (*p* = 0.150), prochlorperazine (*p* = 0.170), and isoniazid (*p* = 0.282).

### 3.2 Patients With Chronic Liver Disease

Between April 1, 2000 and April 30, 2023, we identified 17 512 patients with CLD who had a hospitalization for severe ALI. Among these 17 512 patients, we identified 81 190 new dispensings of 543 unique drugs within 365 days prior to a hospitalization for severe ALI in the primary analysis. Of these, 38 048 new dispensings (46.9%) were within the first 133 days. Among the 543 unique drugs screened for possible associations with severe ALI, those identified as alerts were from the following classes (Table 2; Figure 2): diuretics (spironolactone, furosemide [both *p* < 0.001]), antiemetics (ondansetron, metoclopramide, promethazine [all *p* < 0.001]), appetite stimulants (*p* < 0.001), analgesics (morphine, oxycodone, fentanyl [all *p* < 0.001]), chemotherapy (sorafenib [*p* < 0.001]), antibiotics (ciprofloxacin [*p* = 0.011], metronidazole *[p* = 0.020]), an antipsychotic (prochlorperazine [*p* = 0.105]), vitamins (*p* = 0.134), acid‐suppressives (omeprazole [*p* = 0.164]), and gastrointestinal/liver disease treatments (lactulose, senna glycosides, docusate, silicones, antiflatulents [all *p* < 0.001]; sucralfate [*p* = 0.005]; albumin [*p* = 0.228]).

**TABLE 2:** Drugs with *p* < 0.3 in days 1–133 among people with chronic liver disease.

Node	Drug description	Number of severe ALI cases	Observed cases in window	Expected cases in window	Observed/Expected	Observed‐expected	Test statistic	*p*
A06AD11	Lactulose	1951	1170	914.05	1.29	262.21	69.11	0.001
C03DA01	Spironolactone	2260	1319	1058.81	1.25	267.58	61.81	0.001
C03CA01	Furosemide	2629	1486	1231.69	1.21	262.76	50.94	0.001
A06AB06	Senna glycosides	792	505	371.05	1.37	135.26	46.16	0.001
N02AA01	Morphine	652	418	305.46	1.37	113.44	39.54	0.001
A15zzzz	Appetite stimulants	213	154	99.79	1.55	54.35	28.44	0.001
A04AA01	Ondansetron	1200	690	562.20	1.23	129.70	27.70	0.001
N02AA05	Oxycodone	1198	685	561.26	1.22	125.58	26.00	0.001
A06AB56	Senna glycosides~combinations	396	237	185.53	1.28	51.72	13.51	0.001
L01EX02	Sorafenib	171	113	80.11	1.41	32.96	12.84	0.001
A03FA01	Metoclopramide	229	145	107.29	1.35	37.82	12.56	0.001
A06AA02	Docusate	1250	671	585.63	1.15	86.70	11.86	0.001
A12BA01	Potassium chloride	1534	809	718.68	1.13	92.05	10.85	0.001
N02AB03	Fentanyl	121	82	56.69	1.45	25.35	10.78	0.001
A03AX13	Silicones	424	246	198.64	1.24	47.60	10.66	0.001
A02AF02	Ordinary salt combinations and antiflatulents	189	120	88.55	1.36	31.53	10.58	0.001
R06AD02	Promethazine	483	275	226.29	1.22	49.00	9.91	0.001
A02BX02	Sucralfate	93	64	43.57	1.47	20.45	9.15	0.005
J01MA02	Ciprofloxacin	1521	792	712.59	1.11	80.91	8.46	0.011
P01AB01	Metronidazole	230	138	107.76	1.28	30.33	8.01	0.020
N05AB04	Prochlorperazine	614	332	287.66	1.16	44.68	6.46	0.105
V06DBzz	Fat/carbohydrates/proteins/minerals/vitamins~combinations	156	95	73.09	1.30	21.96	6.20	0.134
A02BC01	Omeprazole	2458	1236	1151.58	1.08	87.04	5.99	0.164
B05AA01	Albumin	30	23	14.06	1.64	8.95	5.57	0.228

Sensitivity analyses conducted after pruning medications in ATC branch group A (except for branch group A02, acid‐suppressive drugs; Table S7) additionally identified alerts for other combinations of nutrients (*p* = 0.174), gatifloxacin (*p* = 0.250), and chlorpromazine (*p* = 0.277). The sensitivity analysis evaluating a shorter risk window of 1–84 days (Table S8) additionally identified alerts for nystatin (*p* = 0.024), ursodiol (*p* = 0.075), and dronabinol (*p* = 0.143). The sensitivity analysis evaluating a risk window of 1–28 days (Table S9) additionally identified dronabinol [*p* < 0.001], nystatin (*p* = 0.006), dalteparin (*p* = 0.018), other combinations of nutrients (*p* = 0.078), dicyclomine (*p* = 0.114), and ursodiol (*p* = 0.183). When we repeated the analysis after increasing the minimum number of required severe ALI cases for each medication from five to 10 (Table S10), the number of unique drugs evaluated decreased from 543 to 445, but no new alerts were identified.

## 4 Discussion

In this study, we applied the tree‐based scan statistic within US VA data to identify medications possibly associated with hospitalization for severe ALI separately among patients without liver/biliary disease and in those with CLD. These medications could be further investigated within future pharmacoepidemiologic studies.

The use of high‐throughput screening to identify hepatotoxic drugs overcomes existing limitations in hepatotoxicity research, which relies primarily on case reports, by utilizing real‐world data to detect associations between medication exposures and severe ALI in a rapid, efficient manner. Importantly, we evaluated associations between medications and ALI not only among people without CLD but also in those with CLD, which is a population that has been largely excluded from observational studies of drug hepatotoxicity, including drug‐induced ALI registries and networks [35, 36]. The approach employed in this study enables the identification of a more limited number of potentially hepatotoxic drugs that could be evaluated within future studies to evaluate incidence rates of severe ALI, as we recently have shown [37].

Our high‐throughput screening analyses identified several potentially hepatotoxic medications, including several not previously recognized. Antibiotics identified as alerts in this study, including amoxicillin/clavulanate, ciprofloxacin, isoniazid and ethambutol, are known to be associated with hepatotoxicity, lending face validity to our analyses [38]. However, the hepatotoxic potential of metronidazole, particularly among people with CLD, is less clear. Metronidazole is primarily metabolized by the liver, and several cases of metronidazole‐induced severe ALI have also been reported among patients without CLD [39, 40, 41]. Future studies should evaluate the hepatotoxic potential of metronidazole, particularly among people with CLD. Similarly, evaluation of other medications identified as alerts in primary analyses, such as diuretics (spironolactone, furosemide), antiemetics (ondansetron, promethazine), acid‐suppressives (omeprazole, ranitidine), appetite stimulants, analgesics (morphine, oxycodone, fentanyl), antipsychotics (prochlorperazine), and chemotherapeutic agents (pazopanib, sorafenib), should also be examined in additional analyses stratified by CLD status.

To prioritize the alerts identified by high‐throughput screening for evaluation in future pharmacoepidemiologic analyses, some research teams have employed focus groups comprised of a multidisciplinary expert panel [42, 43, 44, 45, 46]. The focus group can: (1) evaluate and rank‐order the alerts from high‐throughput screening—a complex task requiring clinical, pharmacologic, and pharmacoepidemiologic knowledge to consider biologic plausibility, frequency of use, and clinical importance; (2) recommend design parameters (e.g., valid drug comparator, suitable negative control comparator, time‐fixed and time‐varying confounders to adjust for); and (3) suggest prespecified hypotheses, including about likely high‐risk subgroups. This approach can identify mechanistically plausible hepatotoxic drugs [47] and suggest formal hypothesis testing for future analyses.

The high‐throughput screening analyses did identify alerts from a number of medications that may be dispensed as treatments for gastrointestinal/hepatic conditions. These included drugs used to treat nausea (ondansetron, promethazine), constipation (lactulose, senna glycosides, metoclopramide, docusate, silicones), and end‐stage liver disease (albumin, lactulose, sucralfate). Drugs known to be potential treatments for gastrointestinal/hepatic conditions may be detected due to protopathic bias. Since we performed conditional analyses using the tree‐based scan statistic, comparing each drug of interest to all other drugs, inclusion of potential treatments for severe ALI in analyses may mask important associations between other drugs and severe ALI. Consequently, we performed sensitivity analyses excluding such medications from the tree of drugs to be examined to identify alerts with other medications. Additionally, among patients with CLD, we identified alerts for medication used to treat decompensated cirrhosis, including diuretics (spironolactone, furosemide) and albumin for the treatment of ascites and lactulose for the treatment of hepatic encephalopathy. This highlights the importance of a clinical assessment of alerts to consider which drugs might be used as therapies for the health outcome under study.

Our study had several potential limitations. First, there is the potential for misclassification of hospitalization for severe ALI events. Our use of inpatient laboratory tests recorded within the first 2 days of hospitalization admission to identify severe ALI events minimizes the likelihood of misclassification of this outcome. A prior analysis showed that 76% of the hospitalizations for severe ALI among patients without CLD were classified as drug‐related by hepatologist review [10], but patients with CLD were not included. To increase the likelihood that severe ALI events were medication‐related, we excluded patients with diagnoses of pancreaticobiliary disease or other conditions that might precipitate findings consistent with severe ALI. Second, it is possible that concomitantly used hepatotoxic medications might have additive effects on associations with severe ALI, but it was beyond the scope of our analyses to examine associations between specific combinations of drugs and these outcomes. Third, tree‐based scan statistics adjust for the multiple drugs being assessed [11, 17]. While adjusting for multiplicity reduces the type I error rate, this approach lowers the sensitivity of our screening, possibly resulting in the dismissal of potentially important signals. Fourth, our study samples were comprised of mostly male patients. However, although patients in the VA are predominantly male, the total sample of women in care in the VA system (~500 000 women) is as large as in many major cohort studies [48, 49]. Finally, we did not confirm the alerts identified in the VA data within another external database. Future studies should seek to replicate the signals identified in this study within other data sources.

In conclusion, high‐throughput pharmacoepidemiologic screening of medications within the real‐world data of the VA system using the tree‐based scan statistic identified potentially hepatotoxic medications that could be further investigated in future pharmacoepidemiologic studies. Our approach could transform hepatic drug safety research by establishing hypothesis‐generating screening studies as a new approach to enhance pharmacovigilance for hepatotoxicity.

### 4.1 Plain Language Summary

Drugs associated with acute liver injury (ALI) are primarily identified by case reports. High‐throughput screening of real‐world data is a novel method that could enable detection of drugs potentially associated with hepatotoxicity. We performed high‐throughput screening of US Veterans Health Administration data using the tree‐based scan statistic to identify drugs possibly associated with hospitalization for severe ALI separately among patients without liver/biliary disease and in those with chronic liver disease (CLD). Among 12 860 patients without liver/biliary disease hospitalized for severe ALI, we evaluated associations with 450 newly dispensed drugs within 365 days prior to hospitalization. Drugs associated with severe ALI were: antiemetics (ondansetron, promethazine), acid‐suppressives (ranitidine, omeprazole), antibiotics (amoxicillin/clavulanate, ciprofloxacin, mupirocin, ethambutol), anticoagulants (heparin), and chemotherapy (pazopanib). Among 17 512 patients with CLD hospitalized for severe ALI, we examined associations with 543 newly dispensed drugs within 365 days prior to hospitalization. Drugs associated with severe ALI were: diuretics (spironolactone, furosemide), antiemetics (ondansetron, metoclopramide, promethazine), appetite stimulants, analgesics (morphine, oxycodone, fentanyl), chemotherapy (sorafenib), antibiotics (ciprofloxacin, metronidazole), antipsychotics (prochlorperazine), vitamins, acid‐suppressives (omeprazole), and gastrointestinal/liver disease treatments (lactulose, senna, docusate, silicones, antiflatulents, sucralfate, albumin). Our analyses demonstrate that high‐throughput screening using tree‐based scan statistics can detect potentially hepatotoxic drugs for investigation in future pharmacoepidemiology studies.

## Funding

This work was supported by the the National Cancer Institute (R01CA206465) and the National Institute on Alcohol Abuse and Alcoholism (P01 AA029545, U24AA020794, U01AA020790, U24AA022001, U01AA013566).

## Ethics Statement

This study was approved by the Institutional Review Boards of the University of Pennsylvania, Yale University, and VA Connecticut Healthcare System.

## Conflicts of Interest

C.E.L. has recently received honoraria from the US National Institutes of Health and the American College of Clinical Pharmacy Foundation. He consults for Moderna, TriNetX, and Shine Lawyers. His spouse is employed by Merck; neither C.E.L. nor his spouse owns stock in Merck. The other authors declare no conflicts of interest.

## Supporting information


**Data S1:** Supporting Information.
