# Supplementary material for: High‐Throughput Screening Using the Self‐Controlled Tree‐Based Scan Statistic to Identify Medications Associated With Hospitalization for Severe Acute Liver Injury
Source: Pharmacoepidemiol Drug Saf. 2025 Dec 4;34(12):e70275. doi: 10.1002/pds.70275 (PMC12678845; doi:10.1002/pds.70275)
Supplement: Supplementary file 1 — Data S1: Supporting Information. [file PDS-34-e70275-s001.docx]

**Supplementary Appendix**

**High-throughput screening using the self-controlled tree-based scan statistic to identify medications potentially associated with hospitalization for severe acute liver injury**

Vincent Lo Re III, Craig W. Newcomb, Dean M. Carbonari, Charles E. Leonard, Christopher T. Rentsch, Judith C. Maro

[**Supplemental Statistical Appendix 1.** Bernoulli Tree-Based Scan Statistics. 3](#_Toc212482933)

[**Supplemental Statistical Appendix 2**. Adjustment for Multiple Testing. 6](#_Toc212482934)

[**Table S1**. List of International Classification of Diseases, Ninth Revision (ICD-9) and International Classification of Diseases, Tenth Revision (ICD-10) diagnostic codes used to identify incident liver/biliary disease or other condition precipitating findings of acute liver injury for exclusion and censoring. Five diagnostic codes in the other unspecified hepatitis/liver disease category were not used for censoring because of the potential for clinicians to record drug-induced liver injury with these codes. 9](#_Toc212482935)

[**Table S2**. List of International Classification of Diseases, Ninth Revision (ICD-9) and International Classification of Diseases, Tenth Revision (ICD-10) diagnostic codes used to identify chronic liver disease. 26](#_Toc212482936)

[**Table S3**. Drugs with p<0.3 in Days 1-133 excluding all drugs with an Anatomical Therapeutic Chemical (ATC) code starting with “A” (i.e., alimentary tract and metabolism) except for “A02” (i.e., drugs for acid related disorders) among people without chronic liver disease. 31](#_Toc212482937)

[**Table S4**. Drugs with p<0.3 in Days 1-84 among people without chronic liver disease. 32](#_Toc212482938)

[**Table S5**. Drugs with p<0.3 in Days 1-28 among people without chronic liver disease. 33](#_Toc212482939)

[**Table S6**. Drugs with p<0.3 in Days 1-133 among people without chronic liver disease in a sensitivity analysis increasing the minimum number of observed cases from 5 to 10. 34](#_Toc212482940)

[**Table S7**. Drugs with p<0.3 in Days 1-133 excluding all drugs with an Anatomical Therapeutic Chemical code starting with “A” (i.e., alimentary tract and metabolism) except for “A02” (i.e., drugs for acid related disorders) among people with chronic liver disease. 35](#_Toc212482941)

[**Table S8**. Drugs with p<0.3 in Days 1-84 among people with chronic liver disease. 36](#_Toc212482942)

[**Table S9**. Drugs with p<0.3 in Days 1-28 among people with chronic liver disease. 37](#_Toc212482943)

[**Table S10**. Drugs with p<0.3 in Days 1-133 among people with chronic liver disease in a sensitivity analysis increasing the minimum number of observed cases from 5 to 10. 38](#_Toc212482944)

# **Supplemental Statistical Appendix 1.** Bernoulli Tree-Based Scan Statistics.

Bernoulli probability models are classically associated with “coin flips” where there is an outcome of 1 or 0 (i.e., heads or tails) with some underlying probability (i.e., 50% if the coin is unweighted). When there is more than 1 coin flip and the probability is unchanged over subsequent trials (i.e., independent and identically distributed), the overall distribution is binomial.

In the self-controlled design employed in this study, we identify all the hospitalized severe acute liver injury (ALI) cases and all the qualifying antecedent drug dispensings. Then, for each incident drug dispensing separately, we model the time-to-event of the severe ALI occurrence as a coin flip with the outcome belonging to 1 of 2 groups (i.e., ALI occurs in the designated risk window after the identified drug dispensing or ALI occurs in the comparison window after the identified drug dispensing) with some Bernoulli probability. For example, in our study, in **Table 1**, there are 286 people experiencing severe ALI events following incident ondansetron dispensings. We model whether the time-to-event from ondansetron to the severe ALI event is within the risk window (in **Table 1**, days 1-133) or in the comparison window (in **Table 1**, days 134-365). If we were to model the Bernoulli probability strictly on the time contributed (i.e., a uniform distribution), then the Bernoulli probability would be (133 days/365 days) or 0.36. In other words, the underlying assumption is that the severe ALI following ondansetron dispensing is equally likely to occur on any day in the 365-day observation window. This is referred to as the “unconditional” Bernoulli tree-based scan statistic within the TreeScan^TM^ software.

The “conditional” Bernoulli tree-based scan statistic used in this study is reserved for self-controlled designs where the underlying probability model over the observation window of 365 days is additionally adjusted. Under this model, the probability is assumed to be non-uniform due to a heightened period of increased monitoring (i.e., differential detection of an event) immediately following new drug dispensing. This phenomenon is assumed to be global across all new drug dispensings rather than following any particular drug dispensing. In other words, in this model, the lengths of the two windows are ignored, and instead the Bernoulli probability is based on the proportion of the sum of outcomes in the risk window compared to the total number of outcomes observed at any time. These conditional analyses attenuate the effect of near-term differential detection capability following incident drug dispensings.

For this analysis, a “node” in the following formulas is a unique drug product or drug class.

The log likelihood ratio (LLR) is derived from a Binomial-based maximum likelihood estimator and is defined as:

$$LLR=\ln\left( \frac{\left( \frac{c_{G}}{c_{G}+n_{G}} \right)^{c_{G}}\left( \frac{n_{G}}{c_{G}+n_{G}} \right)^{n_{G}}}{\left( \frac{C}{C+N} \right)^{c_{G}}\left( \frac{N}{C+N} \right)^{n_{G}}} \right)I\left( \frac{c_{G}}{c_{G}+n_{G}} >\frac{C}{C+N} \right)$$

*c_G_* = the number of outcomes in the node *G* of interest that are also in the risk window

*n_G_* = the number of outcomes in the node that are NOT in the risk window

*C* = the total number of outcomes in the risk window for all nodes

*N* = the total number of outcomes NOT in the risk window for all nodes

*I()* is the indication function, which is 1 when there are more outcomes in the risk window than expected under the null hypothesis, and it is included to ensure that we are looking for an excess risk of having the adverse event rather than a protective decreased risk.

Log likelihood ratios are used for computational convenience as opposed to likelihood ratios. The order in which the nodes are evaluated does not impact the results. The node *G* with the maximum LLR is the most likely cluster of unexplained outcomes in the risk window and its log likelihood ratio is the test statistic:

The distribution of *T* is not known analytically, and so inference is conducted using Monte Carlo hypothesis testing with data permutation on the original “real” dataset. First, a user-defined number of simulated random datasets (e.g., 99,999) are generated under the null hypothesis with the same *C* and *N* as the real data*,* so that the total number of outcomes in the risk window and control windows are the same in both the real and all the random datasets. *T* is calculated for the 99,999 random data sets and the one real dataset.

If the *T* in the real data are among the 5% highest of all the maxima from the real and 99,999 random datasets generated under the null hypothesis, then that node constitutes a signal at the alpha=0.05 statistical significance level. The Monte Carlo based p-value is calculated as *p=R/(99999 + 1)*, where *R* is the rank of the *T* in the real dataset in relation to the *T* in the random datasets. That way, the method formally adjusts the p-values for the multiple scenarios generated by any overlapping groupings. This means that, when the composite null hypothesis is true, there is a 95% probability that all p-values are greater than 0.05, or in other words, that there is not a single exposure-outcome pair or grouping with p≤0.05.

The number of expected cases in the risk window is given by the following formula:

$$\left( c_{G}+ n_{G} \right)* \frac{C}{C+N}$$

# **Supplemental Statistical Appendix 2**. Adjustment for Multiple Testing.

The tree-based scan statistic is a data-mining method that adjusts for multiple testing of correlated hypotheses when screening hundreds of medications for an adverse event of interest for the purpose of signal identification.^1,2^ The tree-based scan statistic detects clusters that cannot be explained by random chance alone.^3^ Detection of these clusters occurs by using maximum likelihood estimation of a generalized likelihood ratio test, which is specifically used for testing a composite hypothesis.^4^ In the present study, we have a composite null hypothesis (rather than a simple binary hypothesis) because in our scenario there may be many medications with an excess of occurrences of severe ALI during the time intervals of interest. Generally, there is no analytically tractable solution to generate a test statistic with a composite null hypothesis. As a result, we use Monte Carlo simulation to create an exact test because we can simulate a null distribution of the test statistic using data perturbation.^5^

We used a data perturbation process to generate 999 simulated random datasets under the composite null hypothesis that no medication exposure is associated with an excess of severe ALI events during the 133 days following exposure compared with the overall 365-day observation window. Each simulated random dataset resulted in a maximum log-likelihood ratio across all medication exposures (i.e., we took the log-likelihood of each medication exposure and only kept the maximum, which is the most extreme departure from the null). Using the maximum is the key to the multiplicity adjustment across the drug exposures.

Below is a distribution of the maximum log-likelihood ratios (1 per 999 simulated random datasets) in the analysis among individuals without any history of liver or biliary disease. If we use a conventional statistical significance value of p<0.05 (one-sided), then we set the threshold when the maximum log-likelihood ratio is ≥7.08 in these data and is illustrated in the figure below. We then compare the log-likelihood ratio in our real data for each medication exposure to this distribution of the maximum log-likelihood ratios. If the log-likelihood ratio on an individual medication exposure exceeds 7.08, then we will declare that there is a statistically significant departure from the composite null hypothesis, since all the values in the figure below were obtained under the conditions when the composite null hypothesis was true. For each medication exposure in our analysis where we calculate a log-likelihood ratio, we will assign it the empirical p-value according to the figure below. In this way, the same composite null hypothesis test produces multiplicity-adjusted p-values for all of the medication exposures in our analysis. The largest departure from the composite null hypothesis in this analysis was ondansetron, which had a log-likelihood ratio of 19.13 (which is outside of the range of observed maximum log-likelihood ratios in all of the 999 simulated random datasets). Contrast that result with promethazine, which has a log-likelihood ratio of 6.66, a value associated with the 93^rd^ percentile in the figure below. Thus, roughly 7% of the time, the maximum log likelihood ratio in simulated null datasets was higher. We have less evidence to suggest that promethazine exhibits unusual behavior vis-à-vis expectation as compared to ondansetron.


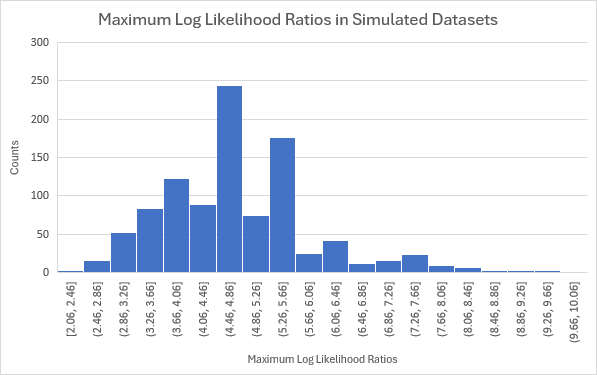


95th Percentile, 7.08

This distribution is analogous to the way that a standard normal distribution is used to assign Z-scores. When performing a one-sided test, the upper portion of the distribution (or the upper and lower portions of the distribution if doing a two-sided test) are the most extreme parts of the distribution. This is the equivalent of saying that if a Z-score measure is >1.96, it has a statistical departure from the expected value in the normal distribution.

If we performed this analysis with multiple hypothesis tests, then we would not use the *maximum* log-likelihood ratio across all medications as the test statistic. Rather, we would test medication by medication (perhaps in a loop) and then use a Bonferroni (or other false discovery rate) correction. In that circumstance, the p-values would not be implicitly multiplicity-adjusted in the way they are in this analysis. This approach was chosen generally because of the high degree of overlap in measurements when we look at related medications in the tree hierarchy. We note in this analysis that we do not ultimately make use of that hierarchy, but we retain the approach nonetheless.

**REFERENCES**

1. Kulldorff M, Fang Z, Walsh SJ. A tree-based scan statistic for database disease surveillance. *Biometrics*. Jun 2003;59(2):323-31. doi:10.1111/1541-0420.00039

2. Kulldorff M, Dashevsky I, Avery TR, et al. Drug safety data mining with a tree-based scan statistic. *Pharmacoepidemiol Drug Saf*. May 2013;22(5):517-23. doi:10.1002/pds.3423

3. Naus JI. The distribution of the size of the maximum cluster of points on a line. *J Am Stat Assoc*. 1965;60(310):532-538.

4. Randle DH, Wolfe DA. Introduction to the Theory of Nonparametric Statistics. Wiley, New York (1979).

5. Dwass M. Modified randomization tests for nonparametric hypotheses. *Ann Math Statist*. 1957;28(1):181-187.

# **Table S1**. List of International Classification of Diseases, Ninth Revision (ICD-9) and International Classification of Diseases, Tenth Revision (ICD-10) diagnostic codes used to identify incident liver/biliary disease or other condition precipitating findings of acute liver injury for exclusion and censoring. Five diagnostic codes in the other unspecified hepatitis/liver disease category were not used for censoring because of the potential for clinicians to record drug-induced liver injury with these codes.

| **Type of Condition** | **Code Type** | **Code** | **Description** |
| --- | --- | --- | --- |
| Acquired hemolytic anemia | ICD-9 | 283.1 | Non-autoimmune hemolytic anemia |
| Acquired hemolytic anemia | ICD-9 | 283.10 | Non-autoimmune hemolytic anemia, unspecified |
| Acquired hemolytic anemia | ICD-9 | 283.11 | Hemolytic uremic syndrome |
| Acquired hemolytic anemia | ICD-9 | 283.19 | Other non-autoimmune hemolytic anemias |
| Acquired hemolytic anemia | ICD-9 | 283.2 | Hemoglobinuria due to hemolysis from external causes |
| Acquired hemolytic anemia | ICD-9 | 283.9 | Acquired hemolytic anemia, unspecified |
| Acquired hemolytic anemia | ICD-10 | D59.1 | Other autoimmune hemolytic anemias |
| Acquired hemolytic anemia | ICD-10 | D59.10 | Autoimmune hemolytic anemia, unspecified |
| Acquired hemolytic anemia | ICD-10 | D59.11 | Warm autoimmune hemolytic anemia |
| Acquired hemolytic anemia | ICD-10 | D59.12 | Cold autoimmune hemolytic anemia |
| Acquired hemolytic anemia | ICD-10 | D59.13 | Mixed type autoimmune hemolytic anemia |
| Acquired hemolytic anemia | ICD-10 | D59.19 | Other autoimmune hemolytic anemia |
| Acquired hemolytic anemia | ICD-10 | D59.3 | Hemolytic-uremic syndrome |
| Acquired hemolytic anemia | ICD-10 | D59.30 | Hemolytic-uremic syndrome, unspecified |
| Acquired hemolytic anemia | ICD-10 | D59.31 | Infection-associated hemolytic-uremic syndrome |
| Acquired hemolytic anemia | ICD-10 | D59.32 | Hereditary hemolytic-uremic syndrome |
| Acquired hemolytic anemia | ICD-10 | D59.39 | Other hemolytic-uremic syndrome |
| Acquired hemolytic anemia | ICD-10 | D59.4 | Other nonautoimmune hemolytic anemias |
| Acquired hemolytic anemia | ICD-10 | D59.5 | Paroxysmal nocturnal hemoglobinuria [Marchiafava-Micheli] |
| Acquired hemolytic anemia | ICD-10 | D59.6 | Hemoglobinuria due to hemolysis from other external causes |
| Acquired hemolytic anemia | ICD-10 | D59.8 | Other acquired hemolytic anemias |
| Acquired hemolytic anemia | ICD-10 | D59.9 | Acquired hemolytic anemia, unspecified |
| Anemia due to enzyme disorders | ICD-9 | 282.2 | Anemias due to disorders of glutathione metabolism |
| Anemia due to enzyme disorders | ICD-9 | 282.3 | Other hemolytic anemias due to enzyme deficiency |
| Anemia due to enzyme disorders | ICD-10 | D55 | Anemia due to enzyme disorders |
| Anemia due to enzyme disorders | ICD-10 | D55.0 | Anemia due to glucose-6-phosphate dehydrogenase [G6PD] deficiency |
| Anemia due to enzyme disorders | ICD-10 | D55.1 | Anemia due to other disorders of glutathione metabolism |
| Anemia due to enzyme disorders | ICD-10 | D55.2 | Anemia due to disorders of glycolytic enzymes |
| Anemia due to enzyme disorders | ICD-10 | D55.21 | Anemia due to pyruvate kinase deficiency |
| Anemia due to enzyme disorders | ICD-10 | D55.29 | Anemia due to other disorders of glycolytic enzymes |
| Anemia due to enzyme disorders | ICD-10 | D55.3 | Anemia due to disorders of nucleotide metabolism |
| Anemia due to enzyme disorders | ICD-10 | D55.8 | Other anemias due to enzyme disorders |
| Anemia due to enzyme disorders | ICD-10 | D55.9 | Anemia due to enzyme disorder, unspecified |
| Anemia in chronic diseases | ICD-9 | 285.2 | Anemia of chronic illness |
| Anemia in chronic diseases | ICD-9 | 285.21 | Anemia in chronic kidney disease |
| Anemia in chronic diseases | ICD-9 | 285.22 | Anemia in neoplastic disease |
| Anemia in chronic diseases | ICD-9 | 285.29 | Anemia in other chronic disease |
| Anemia in chronic diseases | ICD-10 | D63 | Anemia in chronic diseases classified elsewhere |
| Anemia in chronic diseases | ICD-10 | D63.0 | Anemia in neoplastic disease |
| Anemia in chronic diseases | ICD-10 | D63.1 | Anemia in chronic kidney disease |
| Anemia in chronic diseases | ICD-10 | D63.8 | Anemia in other chronic diseases classified elsewhere |
| Biliary disease | ICD-9 | 574 | Cholelithiasis |
| Biliary disease | ICD-9 | 574.0 | Calculus of gallbladder with acute cholecystitis |
| Biliary disease | ICD-9 | 574.00 | Calculus of gallbladder with acute cholecystitis, without mention of obstruction |
| Biliary disease | ICD-9 | 574.01 | Calculus of gallbladder with acute cholecystitis, with obstruction |
| Biliary disease | ICD-9 | 574.1 | Calculus of gallbladder with other cholecystitis |
| Biliary disease | ICD-9 | 574.10 | Calculus of gallbladder with other cholecystitis, without mention of obstruction |
| Biliary disease | ICD-9 | 574.11 | Calculus of gallbladder with other cholecystitis, with obstruction |
| Biliary disease | ICD-9 | 574.2 | Calculus of gallbladder without mention of cholecystitis |
| Biliary disease | ICD-9 | 574.20 | Calculus of gallbladder without mention of cholecystitis, without mention of obstruction |
| Biliary disease | ICD-9 | 574.21 | Calculus of gallbladder without mention of cholecystitis, with obstruction |
| Biliary disease | ICD-9 | 574.3 | Calculus of bile duct with acute cholecystitis |
| Biliary disease | ICD-9 | 574.30 | Calculus of bile duct with acute cholecystitis, without mention of obstruction |
| Biliary disease | ICD-9 | 574.31 | Calculus of bile duct with acute cholecystitis, with obstruction |
| Biliary disease | ICD-9 | 574.4 | Calculus of bile duct with other cholecystitis |
| Biliary disease | ICD-9 | 574.40 | Calculus of bile duct with other cholecystitis, without mention of obstruction |
| Biliary disease | ICD-9 | 574.41 | Calculus of bile duct with other cholecystitis, with obstruction |
| Biliary disease | ICD-9 | 574.5 | Calculus of bile duct without mention of cholecystitis |
| Biliary disease | ICD-9 | 574.50 | Calculus of bile duct without mention of cholecystitis, without mention of obstruction |
| Biliary disease | ICD-9 | 574.51 | Calculus of bile duct without mention of cholecystitis, with obstruction |
| Biliary disease | ICD-9 | 574.6 | Calculus of gallbladder and bile duct with acute cholecystitis |
| Biliary disease | ICD-9 | 574.60 | Calculus of gallbladder and bile duct with acute cholecystitis, without mention of obstruction |
| Biliary disease | ICD-9 | 574.61 | Calculus of gallbladder and bile duct with acute cholecystitis, with obstruction |
| Biliary disease | ICD-9 | 574.7 | Calculus of gallbladder and bile duct with other cholecystitis |
| Biliary disease | ICD-9 | 574.70 | Calculus of gallbladder and bile duct with other cholecystitis, without mention of obstruction |
| Biliary disease | ICD-9 | 574.71 | Calculus of gallbladder and bile duct with other cholecystitis, with obstruction |
| Biliary disease | ICD-9 | 574.8 | Calculus of gallbladder and bile duct with acute and chronic cholecystitis |
| Biliary disease | ICD-9 | 574.80 | Calculus of gallbladder and bile duct with acute and chronic cholecystitis, without mention of obstruction |
| Biliary disease | ICD-9 | 574.81 | Calculus of gallbladder and bile duct with acute and chronic cholecystitis, with obstruction |
| Biliary disease | ICD-9 | 574.9 | Calculus of gallbladder and bile duct without cholecystitis |
| Biliary disease | ICD-9 | 574.90 | Calculus of gallbladder and bile duct without cholecystitis, without mention of obstruction |
| Biliary disease | ICD-9 | 574.91 | Calculus of gallbladder and bile duct without cholecystitis, with obstruction |
| Biliary disease | ICD-9 | 575 | Other disorders of gallbladder |
| Biliary disease | ICD-9 | 575.0 | Acute cholesystitis |
| Biliary disease | ICD-9 | 575.1 | Other cholecystitis |
| Biliary disease | ICD-9 | 575.10 | Cholecyctitis, unspecified |
| Biliary disease | ICD-9 | 575.11 | Chronic cholecystitis |
| Biliary disease | ICD-9 | 575.12 | Acute and chronic cholesystitis |
| Biliary disease | ICD-9 | 575.2 | Obstruction of gallbladder |
| Biliary disease | ICD-9 | 575.3 | Hydrops of gallbladder |
| Biliary disease | ICD-9 | 575.4 | Perforation of gallbladder |
| Biliary disease | ICD-9 | 575.5 | Fistula of gallbladder |
| Biliary disease | ICD-9 | 575.9 | Unspecified disorder of gallbladder |
| Biliary disease | ICD-9 | 576.1 | Cholangitis |
| Biliary disease | ICD-9 | 576.2 | Obstruction of bile duct |
| Biliary disease | ICD-9 | 576.3 | Perforation of bile duct |
| Biliary disease | ICD-9 | 576.4 | Fistula of bile duct |
| Biliary disease | ICD-9 | 576.9 | Unspecified disorder of biliary tract |
| Biliary disease | ICD-9 | 575.8 | Other specified disorders of gallbladder |
| Biliary disease | ICD-9 | 576.8 | Other specified disorders of biliary tract |
| Biliary disease | ICD-10 | K80 | Cholelithiasis |
| Biliary disease | ICD-10 | K80.0 | Calculus of gallbladder with acute cholecystitis |
| Biliary disease | ICD-10 | K80.00 | Calculus of gallbladder with acute cholecystitis without obstruction |
| Biliary disease | ICD-10 | K80.01 | Calculus of gallbladder with acute cholecystitis with obstruction |
| Biliary disease | ICD-10 | K80.1 | Calculus of gallbladder with other cholecystitis |
| Biliary disease | ICD-10 | K80.10 | Calculus of gallbladder with chronic cholecystitis without obstruction |
| Biliary disease | ICD-10 | K80.11 | Calculus of gallbladder with chronic cholecystitis with obstruction |
| Biliary disease | ICD-10 | K80.12 | Calculus of gallbladder with acute and chronic cholecystitis without obstruction |
| Biliary disease | ICD-10 | K80.13 | Calculus of gallbladder with acute and chronic cholecystitis with obstruction |
| Biliary disease | ICD-10 | K80.18 | Calculus of gallbladder with other cholecystitis without obstruction |
| Biliary disease | ICD-10 | K80.19 | Calculus of gallbladder with other cholecystitis with obstruction |
| Biliary disease | ICD-10 | K80.2 | Calculus of gallbladder without cholecystitis |
| Biliary disease | ICD-10 | K80.20 | Calculus of gallbladder without cholecystitis without obstruction |
| Biliary disease | ICD-10 | K80.21 | Calculus of gallbladder without cholecystitis with obstruction |
| Biliary disease | ICD-10 | K80.3 | Calculus of bile duct with cholangitis |
| Biliary disease | ICD-10 | K80.30 | Calculus of bile duct with cholangitis unspecified, without obstruction |
| Biliary disease | ICD-10 | K80.31 | Calculus of bile duct with cholangitis unspecified, with obstruction |
| Biliary disease | ICD-10 | K80.32 | Calculus of bile duct with acute cholangitis without obstruction |
| Biliary disease | ICD-10 | K80.33 | Calculus of bile duct with acute cholangitis with obstruction |
| Biliary disease | ICD-10 | K80.34 | Calculus of bile duct with chronic cholangitis without obstruction |
| Biliary disease | ICD-10 | K80.35 | Calculus of bile duct with chronic cholangitis with obstruction |
| Biliary disease | ICD-10 | K80.36 | Calculus of bile duct with acute and chronic cholangitis without obstruction |
| Biliary disease | ICD-10 | K80.37 | Calculus of bile duct with acute and chronic cholangitis with obstruction |
| Biliary disease | ICD-10 | K80.4 | Calculus of bile duct with cholecystitis |
| Biliary disease | ICD-10 | K80.40 | Calculus of bile duct with cholecystitis unspecified, without obstruction |
| Biliary disease | ICD-10 | K80.41 | Calculus of bile duct with cholecystitis unspecified, with obstruction |
| Biliary disease | ICD-10 | K80.42 | Calculus of bile duct with acute cholecystitis without obstruction |
| Biliary disease | ICD-10 | K80.43 | Calculus of bile duct with acute cholecystitis with obstruction |
| Biliary disease | ICD-10 | K80.44 | Calculus of bile duct with chronic cholecystitis without obstruction |
| Biliary disease | ICD-10 | K80.45 | Calculus of bile duct with chronic cholecystitis with obstruction |
| Biliary disease | ICD-10 | K80.46 | Calculus of bile duct with acute and chronic cholecystitis without obstruction |
| Biliary disease | ICD-10 | K80.47 | Calculus of bile duct with acute and chronic cholecystitis with obstruction |
| Biliary disease | ICD-10 | K80.5 | Calculus of bile duct without cholangitis or cholecystitis |
| Biliary disease | ICD-10 | K80.50 | Calculus of bile duct without cholangitis or cholecystitis without obstruction |
| Biliary disease | ICD-10 | K80.51 | Calculus of bile duct without cholangitis or cholecystitis with obstruction |
| Biliary disease | ICD-10 | K80.6 | Calculus of gallbladder and bile duct with cholecystitis |
| Biliary disease | ICD-10 | K80.60 | Calculus of gallbladder and bile duct with cholecystitis unspecified, without obstruction |
| Biliary disease | ICD-10 | K80.61 | Calculus of gallbladder and bile duct with cholecystitis unspecified, with obstruction |
| Biliary disease | ICD-10 | K80.62 | Calculus of gallbladder and bile duct with acute cholecystitis without obstruction |
| Biliary disease | ICD-10 | K80.63 | Calculus of gallbladder and bile duct with acute cholecystitis with obstruction |
| Biliary disease | ICD-10 | K80.64 | Calculus of gallbladder and bile duct with chronic cholecystitis without obstruction |
| Biliary disease | ICD-10 | K80.65 | Calculus of gallbladder and bile duct with chronic cholecystitis with obstruction |
| Biliary disease | ICD-10 | K80.66 | Calculus of gallbladder and bile duct with acute and chronic cholecystitis without obstruction |
| Biliary disease | ICD-10 | K80.67 | Calculus of gallbladder and bile duct with acute and chronic cholecystitis with obstruction |
| Biliary disease | ICD-10 | K80.7 | Calculus of gallbladder and bile duct without cholecystitis |
| Biliary disease | ICD-10 | K80.70 | Calculus of gallbladder and bile duct without cholecystitis without obstruction |
| Biliary disease | ICD-10 | K80.71 | Calculus of gallbladder and bile duct without cholecystitis with obstruction |
| Biliary disease | ICD-10 | K80.8 | Other cholelithiasis |
| Biliary disease | ICD-10 | K80.80 | Other cholelithiasis without obstruction |
| Biliary disease | ICD-10 | K80.81 | Other cholelithiasis with obstruction |
| Biliary disease | ICD-10 | K81 | Cholecystitis |
| Biliary disease | ICD-10 | K81.0 | Acute cholecystitis |
| Biliary disease | ICD-10 | K81.1 | Chronic cholecystitis |
| Biliary disease | ICD-10 | K81.2 | Acute cholecystitis with chronic cholecystitis |
| Biliary disease | ICD-10 | K81.9 | Cholecystitis, unspecified |
| Biliary disease | ICD-10 | K82 | Other diseases of gallbladder |
| Biliary disease | ICD-10 | K82.0 | Obstruction of gallbladder |
| Biliary disease | ICD-10 | K82.1 | Hydrops of gallbladder |
| Biliary disease | ICD-10 | K82.2 | Perforation of gallbladder |
| Biliary disease | ICD-10 | K82.3 | Fistula of gallbladder |
| Biliary disease | ICD-10 | K82.8 | Other specified diseases of gallbladder |
| Biliary disease | ICD-10 | K82.9 | Disease of gallbladder, unspecified |
| Biliary disease | ICD-10 | K82.A | Disorders of gallbladder in diseases classified elsewhere |
| Biliary disease | ICD-10 | K82.A1 | Gangrene of gallbladder in cholecystitis |
| Biliary disease | ICD-10 | K82.A2 | Perforation of gallbladder in cholecystitis |
| Biliary disease | ICD-10 | K83.0 | Cholangitis |
| Biliary disease | ICD-10 | K83.09 | Other cholangitis |
| Biliary disease | ICD-10 | K83.1 | Obstruction of bile duct |
| Biliary disease | ICD-10 | K83.2 | Perforation of bile duct |
| Biliary disease | ICD-10 | K83.3 | Fistula of bile duct |
| Biliary disease | ICD-10 | K83.5 | Biliary cyst |
| Biliary disease | ICD-10 | K83.8 | Other specified diseases of biliary tract |
| Biliary disease | ICD-10 | K83.9 | Disease of biliary tract, unspecified |
| Biliary disease | ICD-10 | K87 | Disorders of gallbladder, biliary tract and pancreas in diseases classified elsewhere |
| Budd Chiari Syndrome | ICD-9 | 453.0 | Budd-Chiari syndrome |
| Budd Chiari Syndrome | ICD-10 | I82.0 | Budd Chiari syndrome |
| Cancer in the liver or biliary tree | ICD-9 | 155 | Malignant neoplasm of liver and intrahepatic bile ducts |
| Cancer in the liver or biliary tree | ICD-9 | 155.0 | Malignant neoplasm of liver, primary |
| Cancer in the liver or biliary tree | ICD-9 | 155.1 | Malignant neoplasm of intrahepatic bile ducts |
| Cancer in the liver or biliary tree | ICD-9 | 155.2 | Malignant neoplasm liver, not specified as primary or secondary |
| Cancer in the liver or biliary tree | ICD-9 | 156 | Malignant neoplasm of gallbladder and extrahepatic bile ducts |
| Cancer in the liver or biliary tree | ICD-9 | 156.0 | Malignant neoplasm of gallbladder |
| Cancer in the liver or biliary tree | ICD-9 | 156.1 | Malignant neoplasm of extrahepatic bile ducts |
| Cancer in the liver or biliary tree | ICD-9 | 156.2 | Malignant neoplasm of ampulla of Vater |
| Cancer in the liver or biliary tree | ICD-9 | 156.8 | Malignant neoplasm of other specified sites of gallbladder and extrahepatic bile ducts |
| Cancer in the liver or biliary tree | ICD-9 | 156.9 | Malignant neoplasm of biliary tract, part unspecified site |
| Cancer in the liver or biliary tree | ICD-9 | 197.7 | Malignant neoplasm of liver, secondary |
| Cancer in the liver or biliary tree | ICD-9 | 209.72 | Secondary neuroendocrine tumor of liver |
| Cancer in the liver or biliary tree | ICD-9 | 230.8 | Carcinoma in situ of liver and biliary system |
| Cancer in the liver or biliary tree | ICD-10 | C22 | Malignant neoplasm of liver and intrahepatic bile ducts |
| Cancer in the liver or biliary tree | ICD-10 | C22.0 | Liver cell carcinoma |
| Cancer in the liver or biliary tree | ICD-10 | C22.1 | Intrahepatic bile duct carcinoma |
| Cancer in the liver or biliary tree | ICD-10 | C22.2 | Hepatoblastoma |
| Cancer in the liver or biliary tree | ICD-10 | C22.3 | Angiosarcoma of liver |
| Cancer in the liver or biliary tree | ICD-10 | C22.4 | Other sarcomas of liver |
| Cancer in the liver or biliary tree | ICD-10 | C22.7 | Other specified carcinomas of liver |
| Cancer in the liver or biliary tree | ICD-10 | C22.8 | Malignant neoplasm of liver, primary, unspecified as to type |
| Cancer in the liver or biliary tree | ICD-10 | C22.9 | Malignant neoplasm liver not specified as primary/secondary |
| Cancer in the liver or biliary tree | ICD-10 | C23 | Malignant neoplasm of gallbladder |
| Cancer in the liver or biliary tree | ICD-10 | C24 | Malignant neoplasm of other and unspecified parts of biliary tract |
| Cancer in the liver or biliary tree | ICD-10 | C24.0 | Malignant neoplasm of extrahepatic bile duct |
| Cancer in the liver or biliary tree | ICD-10 | C24.1 | Malignant neoplasm of ampulla of Vater |
| Cancer in the liver or biliary tree | ICD-10 | C24.8 | Malignant neoplasm of overlapping sites of biliary tract |
| Cancer in the liver or biliary tree | ICD-10 | C24.9 | Malignant neoplasm of biliary tract, unspecified |
| Cancer in the liver or biliary tree | ICD-10 | C78.7 | Secondary malignant neoplasm of liver and intrahepatic bile duct |
| Cancer in the liver or biliary tree | ICD-10 | C7B.02 | Secondary carcinoid tumors of liver |
| Cancer in the liver or biliary tree | ICD-10 | D01.5 | Carcinoma in situ of liver, gallbladder and bile ducts |
| Cancer of the pancreas | ICD-9 | 157 | Malignant neoplasm of pancreas |
| Cancer of the pancreas | ICD-9 | 157.0 | Malignant neoplasm of head of pancreas |
| Cancer of the pancreas | ICD-9 | 157.1 | Malignant neoplasm of body of pancreas |
| Cancer of the pancreas | ICD-9 | 157.2 | Malignant neoplasm of tail of pancreas |
| Cancer of the pancreas | ICD-9 | 157.3 | Malignant neoplasm of pancreatic duct |
| Cancer of the pancreas | ICD-9 | 157.4 | Malignant neoplasm of islets of Langerhans |
| Cancer of the pancreas | ICD-9 | 157.8 | Malignant neoplasm of other specified sites of pancreas |
| Cancer of the pancreas | ICD-9 | 157.9 | Malignant neoplasm of pancreas, part unspecified |
| Cancer of the pancreas | ICD-10 | C25 | Malignant neoplasm of pancreas |
| Cancer of the pancreas | ICD-10 | C25.0 | Malignant neoplasm of head of pancreas |
| Cancer of the pancreas | ICD-10 | C25.1 | Malignant neoplasm of body of pancreas |
| Cancer of the pancreas | ICD-10 | C25.2 | Malignant neoplasm of tail of pancreas |
| Cancer of the pancreas | ICD-10 | C25.3 | Malignant neoplasm of pancreatic duct |
| Cancer of the pancreas | ICD-10 | C25.4 | Malignant neoplasm of endocrine pancreas |
| Cancer of the pancreas | ICD-10 | C25.7 | Malignant neoplasm of other parts of pancreas |
| Cancer of the pancreas | ICD-10 | C25.8 | Malignant neoplasm of overlapping sites of pancreas |
| Cancer of the pancreas | ICD-10 | C25.9 | Malignant neoplasm of pancreas, unspecified |
| Celiac disease | ICD-9 | 579.0 | Celiac disease |
| Celiac disease | ICD-10 | K90.0 | Celiac disease |
| Hepatitis A | ICD-9 | 070.0 | Viral hepatitis A with hepatic coma |
| Hepatitis A | ICD-9 | 070.1 | Viral hepatitis A without mention of hepatic coma |
| Hepatitis A | ICD-10 | B15 | Acute hepatitis A |
| Hepatitis A | ICD-10 | B15.0 | Hepatitis A with hepatic coma |
| Hepatitis A | ICD-10 | B15.9 | Hepatitis A without hepatic coma |
| Hepatitis B | ICD-10 | B16 | Acute hepatitis B |
| Hepatitis B | ICD-10 | B16.2 | Acute hepatitis B without delta-agent with hepatic coma |
| Hepatitis B | ICD-10 | B16.9 | Acute hepatitis B without delta-agent and without hepatic coma |
| Hepatitis B and D | ICD-10 | B16.0 | Acute hepatitis B with delta-agent with hepatic coma |
| Hepatitis B and D | ICD-10 | B16.1 | Acute hepatitis B with delta-agent without hepatic coma |
| Hepatitis B and D | ICD-10 | B17.0 | Acute delta-(super) infection of hepatitis B carrier |
| Hepatitis C | ICD-9 | 070.41 | Acute hepatitis C with hepatic coma |
| Hepatitis C | ICD-9 | 070.51 | Acute hepatitis C without mention of hepatic coma |
| Hepatitis C | ICD-10 | B17.1 | Acute hepatitis C |
| Hepatitis C | ICD-10 | B17.10 | Acute hepatitis C without hepatic coma |
| Hepatitis C | ICD-10 | B17.11 | Acute hepatitis C with hepatic coma |
| Hepatitis E | ICD-9 | 070.43 | Hepatitis E with hepatic coma |
| Hepatitis E | ICD-9 | 070.53 | Hepatitis E without mention of hepatic coma |
| Hepatitis E | ICD-10 | B17.2 | Acute hepatitis E |
| Hypotension | ICD-9 | 458 | Hypotension |
| Hypotension | ICD-9 | 458.0 | Orthostatic hypotension |
| Hypotension | ICD-9 | 458.1 | Chronic hypotension |
| Hypotension | ICD-9 | 458.2 | Iatrogenic hypotension |
| Hypotension | ICD-9 | 458.21 | Hypotension of hemodialysis |
| Hypotension | ICD-9 | 458.29 | Other iatrogenic hypotension |
| Hypotension | ICD-9 | 458.8 | Other specified hypotension |
| Hypotension | ICD-9 | 458.9 | Hypotension, unspecified |
| Hypotension | ICD-10 | I95 | Hypotension |
| Hypotension | ICD-10 | I95.0 | Idiopathic hypotension |
| Hypotension | ICD-10 | I95.1 | Orthostatic hypotension |
| Hypotension | ICD-10 | I95.2 | Hypotension due to drugs |
| Hypotension | ICD-10 | I95.3 | Hypotension of hemodialysis |
| Hypotension | ICD-10 | I95.8 | Other hypotension |
| Hypotension | ICD-10 | I95.81 | Postprocedural hypotension |
| Hypotension | ICD-10 | I95.89 | Other hypotension |
| Hypotension | ICD-10 | I95.9 | Hypotension, unspecified |
| Other acute or unspecified viral hepatitis | ICD-9 | 070 | Viral hepatitis |
| Other acute or unspecified viral hepatitis | ICD-9 | 070.4 | Other specified viral hepatitis with hepatic coma |
| Other acute or unspecified viral hepatitis | ICD-9 | 070.49 | Other specified viral hepatitis with hepatic coma |
| Other acute or unspecified viral hepatitis | ICD-9 | 070.5 | Other specified viral hepatitis without mention of hepatic coma |
| Other acute or unspecified viral hepatitis | ICD-9 | 070.59 | Other specified viral hepatitis without mention of hepatic coma |
| Other acute or unspecified viral hepatitis | ICD-9 | 070.6 | Unspecified viral hepatitis with hepatic coma |
| Other acute or unspecified viral hepatitis | ICD-9 | 070.9 | Unspecified viral hepatitis without mention of hepatic coma |
| Other acute or unspecified viral hepatitis | ICD-9 | 072.71 | Mumps hepatitis |
| Other acute or unspecified viral hepatitis | ICD-9 | 573.1 | Hepatitis in viral diseases classified elsewhere |
| Other acute or unspecified viral hepatitis | ICD-9 | V02.69 | Other viral hepatitis carrier |
| Other acute or unspecified viral hepatitis | ICD-10 | B00.81 | Herpesviral hepatitis |
| Other acute or unspecified viral hepatitis | ICD-10 | B17 | Other acute viral hepatitis |
| Other acute or unspecified viral hepatitis | ICD-10 | B17.8 | Other specified acute viral hepatitis |
| Other acute or unspecified viral hepatitis | ICD-10 | B17.9 | Acute viral hepatitis, unspecified |
| Other acute or unspecified viral hepatitis | ICD-10 | B19 | Unspecified viral hepatitis |
| Other acute or unspecified viral hepatitis | ICD-10 | B19.0 | Unspecified viral hepatitis with hepatic coma |
| Other acute or unspecified viral hepatitis | ICD-10 | B19.9 | Unspecified viral hepatitis without hepatic coma |
| Other acute or unspecified viral hepatitis | ICD-10 | B25.1 | Cytomegaloviral hepatitis |
| Other acute or unspecified viral hepatitis | ICD-10 | B26.81 | Mumps hepatitis |
| Other acute or unspecified viral hepatitis | ICD-10 | B94.2 | Sequelae of viral hepatitis |
| Other acute or unspecified viral hepatitis | ICD-10 | O98.4 | Viral hepatitis complicating pregnancy, childbirth and the puerperium |
| Other acute or unspecified viral hepatitis | ICD-10 | O98.41 | Viral hepatitis complicating pregnancy |
| Other acute or unspecified viral hepatitis | ICD-10 | O98.411 | Viral hepatitis complicating pregnancy, first trimester |
| Other acute or unspecified viral hepatitis | ICD-10 | O98.412 | Viral hepatitis complicating pregnancy, second trimester |
| Other acute or unspecified viral hepatitis | ICD-10 | O98.413 | Viral hepatitis complicating pregnancy, third trimester |
| Other acute or unspecified viral hepatitis | ICD-10 | O98.419 | Viral hepatitis complicating pregnancy, unspecified trimester |
| Other acute or unspecified viral hepatitis | ICD-10 | O98.42 | Viral hepatitis complicating childbirth |
| Other acute or unspecified viral hepatitis | ICD-10 | O98.43 | Viral hepatitis complicating the puerperium |
| Other acute or unspecified viral hepatitis | ICD-10 | P35.3 | Congenital viral hepatitis |
| Other hereditary hemolytic anemias | ICD-9 | 282 | Hereditary hemolytic anemias |
| Other hereditary hemolytic anemias | ICD-9 | 282.0 | Hereditary spherocytosis |
| Other hereditary hemolytic anemias | ICD-9 | 282.1 | Hereditary elliptocytosis |
| Other hereditary hemolytic anemias | ICD-9 | 282.7 | Other hemoglobinopathies |
| Other hereditary hemolytic anemias | ICD-9 | 282.8 | Other specified hereditary hemolytic anemias |
| Other hereditary hemolytic anemias | ICD-9 | 282.9 | Hereditary hemolytic anemia, unspecified |
| Other hereditary hemolytic anemias | ICD-10 | D58 | Other hereditary hemolytic anemias |
| Other hereditary hemolytic anemias | ICD-10 | D58.0 | Hereditary spherocytosis |
| Other hereditary hemolytic anemias | ICD-10 | D58.1 | Hereditary elliptocytosis |
| Other hereditary hemolytic anemias | ICD-10 | D58.2 | Other hemoglobinopathies |
| Other hereditary hemolytic anemias | ICD-10 | D58.8 | Other specified hereditary hemolytic anemias |
| Other hereditary hemolytic anemias | ICD-10 | D58.9 | Hereditary hemolytic anemia, unspecified |
| Other unspecified hepatitis/liver disease | ICD-9 | 091.62 | Secondary syphilitic hepatitis |
| Other unspecified hepatitis/liver disease | ICD-9 | 130.5 | Hepatitis due to toxoplasmosis |
| Other unspecified hepatitis/liver disease | ICD-9 | 573.2 | Hepatitis in other infectious diseases classified elsewhere |
| Other unspecified hepatitis/liver disease | ICD-9 | 573.3 | Hepatitis, unspecified |
| Other unspecified hepatitis/liver disease | ICD-9 | 573.4 | Hepatic infarction |
| Other unspecified hepatitis/liver disease | ICD-9 | 573.8 | Other specified disorder of liver |
| Other unspecified hepatitis/liver disease | ICD-9 | 573.9 | Unspecified disorder of liver |
| Other unspecified hepatitis/liver disease | ICD-10 | A51.45 | Secondary syphilitic hepatitis |
| Other unspecified hepatitis/liver disease | ICD-10 | B58.1 | Toxoplasma hepatitis |
| Other unspecified hepatitis/liver disease | ICD-10 | K76.89 | Other specified diseases of liver |
| Other unspecified hepatitis/liver disease | ICD-10 | K76.9 | Liver disease, unspecified |
| Pancreatitis | ICD-9 | 577 | Diseases of pancreas |
| Pancreatitis | ICD-9 | 577.0 | Acute pancreatitis |
| Pancreatitis | ICD-9 | 577.1 | Chronic pancreatitis |
| Pancreatitis | ICD-9 | 577.2 | Cyst and pseudocyst of pancreas |
| Pancreatitis | ICD-9 | 577.9 | Unspecified disease of pancreas |
| Pancreatitis | ICD-9 | 577.8 | Other specified diseases of pancreas |
| Pancreatitis | ICD-10 | K85.0 | Idiopathic acute pancreatitis |
| Pancreatitis | ICD-10 | K85.00 | Idiopathic acute pancreatitis without necrosis or infection |
| Pancreatitis | ICD-10 | K85.01 | Idiopathic acute pancreatitis with uninfected necrosis |
| Pancreatitis | ICD-10 | K85.02 | Idiopathic acute pancreatitis with infected necrosis |
| Pancreatitis | ICD-10 | K85.1 | Biliary acute pancreatitis |
| Pancreatitis | ICD-10 | K85.10 | Biliary acute pancreatitis without necrosis or infection |
| Pancreatitis | ICD-10 | K85.11 | Biliary acute pancreatitis with uninfected necrosis |
| Pancreatitis | ICD-10 | K85.12 | Biliary acute pancreatitis with infected necrosis |
| Pancreatitis | ICD-10 | K85.2 | Alcohol induced acute pancreatitis |
| Pancreatitis | ICD-10 | K85.20 | Alcohol induced acute pancreatitis without necrosis or infection |
| Pancreatitis | ICD-10 | K85.21 | Alcohol induced acute pancreatitis with uninfected necrosis |
| Pancreatitis | ICD-10 | K85.22 | Alcohol induced acute pancreatitis with infected necrosis |
| Pancreatitis | ICD-10 | K85.8 | Other acute pancreatitis |
| Pancreatitis | ICD-10 | K85.80 | Other acute pancreatitis without necrosis or infection |
| Pancreatitis | ICD-10 | K85.81 | Other acute pancreatitis with uninfected necrosis |
| Pancreatitis | ICD-10 | K85.82 | Other acute pancreatitis with infected necrosis |
| Pancreatitis | ICD-10 | K85.9 | Acute pancreatitis, unspecified |
| Pancreatitis | ICD-10 | K85.90 | Acute pancreatitis without necrosis or infection, unspecified |
| Pancreatitis | ICD-10 | K85.91 | Acute pancreatitis with uninfected necrosis, unspecified |
| Pancreatitis | ICD-10 | K85.92 | Acute pancreatitis with infected necrosis, unspecified |
| Pancreatitis | ICD-10 | K86 | Other diseases of pancreas |
| Pancreatitis | ICD-10 | K86.0 | Alcohol-induced chronic pancreatitis |
| Pancreatitis | ICD-10 | K86.1 | Other chronic pancreatitis |
| Pancreatitis | ICD-10 | K86.2 | Cyst of pancreas |
| Pancreatitis | ICD-10 | K86.3 | Pseudocyst of pancreas |
| Pancreatitis | ICD-10 | K86.8 | Other specified diseases of pancreas |
| Pancreatitis | ICD-10 | K86.81 | Exocrine pancreatic insufficiency |
| Pancreatitis | ICD-10 | K86.89 | Other specified diseases of pancreas |
| Pancreatitis | ICD-10 | K86.9 | Disease of pancreas, unspecified |
| Primary sclerosing cholangitis | ICD-10 | K83.01 | Primary sclerosing cholangitis |
| Rhabdomyolysis | ICD-9 | 728.88 | Rhabdomyolysis |
| Rhabdomyolysis | ICD-10 | M62.82 | Rhabdomyolysis |
| Rhabdomyolysis | ICD-10 | T79.6 | Traumatic ischemia of the muscle |
| Rhabdomyolysis | ICD-10 | T79.6XXA | Traumatic ischemia of the muscle initial encounter |
| Rhabdomyolysis | ICD-10 | T79.6XXD | Traumatic ischemia of the muscle subsequent encounter |
| Rhabdomyolysis | ICD-10 | T79.6XXS | Traumatic ischemia of the muscle sequela |
| Sepsis without shock | ICD-9 | 003.1 | Salmonella septicemia |
| Sepsis without shock | ICD-9 | 020.2 | Septicemic plague |
| Sepsis without shock | ICD-9 | 022.3 | Anthrax septicemia |
| Sepsis without shock | ICD-9 | 038 | Septicemia |
| Sepsis without shock | ICD-9 | 038.0 | Streptococcal septicemia |
| Sepsis without shock | ICD-9 | 038.1 | Staphylococcal septicemia |
| Sepsis without shock | ICD-9 | 038.10 | Staphylococcal septicemia, unspecified |
| Sepsis without shock | ICD-9 | 038.11 | Methicillin susceptible Staphylococcus aureus septicemia |
| Sepsis without shock | ICD-9 | 038.12 | Methicillin resistant Staphylococcus aureus septicemia |
| Sepsis without shock | ICD-9 | 038.19 | Other staphylococcal septicemia |
| Sepsis without shock | ICD-9 | 038.2 | Pneumococcal septicemia [Streptococcus pneumoniae septicemia] |
| Sepsis without shock | ICD-9 | 038.3 | Septicemia due to anaerobes |
| Sepsis without shock | ICD-9 | 038.4 | Septicemia due to other gram-negative organisms |
| Sepsis without shock | ICD-9 | 038.40 | Septicemia due to gram-negative organism, unspecified |
| Sepsis without shock | ICD-9 | 038.41 | Septicemia due to hemophilus influenzae [H. influenzae] |
| Sepsis without shock | ICD-9 | 038.42 | Septicemia due to escherichia coli [E. coli] |
| Sepsis without shock | ICD-9 | 038.43 | Septicemia due to pseudomonas |
| Sepsis without shock | ICD-9 | 038.44 | Septicemia due to serratia |
| Sepsis without shock | ICD-9 | 038.49 | Other septicemia due to gram-negative organisms |
| Sepsis without shock | ICD-9 | 038.8 | Other specified septicemias |
| Sepsis without shock | ICD-9 | 038.9 | Unspecified septicemia |
| Sepsis without shock | ICD-9 | 670.2 | Puerperal sepsis |
| Sepsis without shock | ICD-9 | 670.20 | Puerperal sepsis, unspecified as to episode of care or not applicable |
| Sepsis without shock | ICD-9 | 670.22 | Puerperal sepsis, delivered, with mention of postpartum complication |
| Sepsis without shock | ICD-9 | 670.24 | Puerperal sepsis, postpartum condition or complication |
| Sepsis without shock | ICD-9 | 790.7 | Bacteremia |
| Sepsis without shock | ICD-9 | 995.9 | Systemic inflammatory response syndrome (SIRS) |
| Sepsis without shock | ICD-9 | 995.90 | Systemic inflammatory response syndrome, unspecified |
| Sepsis without shock | ICD-9 | 995.91 | Sepsis |
| Sepsis without shock | ICD-9 | 995.92 | Severe sepsis |
| Sepsis without shock | ICD-9 | 995.93 | Systemic inflammatory response syndrome due to noninfectious process without acute organ dysfunction |
| Sepsis without shock | ICD-9 | 995.94 | Systemic inflammatory response syndrome due to noninfectious process with acute organ dysfunction |
| Sepsis without shock | ICD-10 | A02.1 | Salmonella sepsis |
| Sepsis without shock | ICD-10 | A20.7 | Septicemic plague |
| Sepsis without shock | ICD-10 | A22.7 | Anthrax sepsis |
| Sepsis without shock | ICD-10 | A26.7 | Erysipelothrix sepsis |
| Sepsis without shock | ICD-10 | A32.7 | Listerial sepsis |
| Sepsis without shock | ICD-10 | A40 | Streptococcal sepsis |
| Sepsis without shock | ICD-10 | A40.0 | Sepsis due to streptococcus, group A |
| Sepsis without shock | ICD-10 | A40.1 | Sepsis due to streptococcus, group B |
| Sepsis without shock | ICD-10 | A40.3 | Sepsis due to Streptococcus pneumoniae |
| Sepsis without shock | ICD-10 | A40.8 | Other streptococcal sepsis |
| Sepsis without shock | ICD-10 | A40.9 | Streptococcal sepsis, unspecified |
| Sepsis without shock | ICD-10 | A41 | Other sepsis |
| Sepsis without shock | ICD-10 | A41.0 | Sepsis due to Staphylococcus aureus |
| Sepsis without shock | ICD-10 | A41.01 | Sepsis due to Methicillin susceptible Staphylococcus aureus |
| Sepsis without shock | ICD-10 | A41.02 | Sepsis due to Methicillin resistant Staphylococcus aureus |
| Sepsis without shock | ICD-10 | A41.1 | Sepsis due to other specified staphylococcus |
| Sepsis without shock | ICD-10 | A41.2 | Sepsis due to unspecified staphylococcus |
| Sepsis without shock | ICD-10 | A41.3 | Sepsis due to Hemophilus influenzae |
| Sepsis without shock | ICD-10 | A41.4 | Sepsis due to anaerobes |
| Sepsis without shock | ICD-10 | A41.5 | Sepsis due to other Gram-negative organisms |
| Sepsis without shock | ICD-10 | A41.50 | Gram-negative sepsis, unspecified |
| Sepsis without shock | ICD-10 | A41.51 | Sepsis due to Escherichia coli [E. coli] |
| Sepsis without shock | ICD-10 | A41.52 | Sepsis due to Pseudomonas |
| Sepsis without shock | ICD-10 | A41.53 | Sepsis due to Serratia |
| Sepsis without shock | ICD-10 | A41.59 | Other Gram-negative sepsis |
| Sepsis without shock | ICD-10 | A41.8 | Other specified sepsis |
| Sepsis without shock | ICD-10 | A41.81 | Sepsis due to Enterococcus |
| Sepsis without shock | ICD-10 | A41.89 | Other specified sepsis |
| Sepsis without shock | ICD-10 | A41.9 | Sepsis, unspecified organism |
| Sepsis without shock | ICD-10 | A42.7 | Actinomycotic sepsis |
| Sepsis without shock | ICD-10 | A54.86 | Gonococcal sepsis |
| Sepsis without shock | ICD-10 | B37.7 | Candidal sepsis |
| Sepsis without shock | ICD-10 | O03.37 | Sepsis following incomplete spontaneous abortion |
| Sepsis without shock | ICD-10 | O03.87 | Sepsis following complete or unspecified spontaneous abortion |
| Sepsis without shock | ICD-10 | O04.87 | Sepsis following (induced) termination of pregnancy |
| Sepsis without shock | ICD-10 | O07.37 | Sepsis following failed attempted termination of pregnancy |
| Sepsis without shock | ICD-10 | O08.82 | Sepsis following ectopic and molar pregnancy |
| Sepsis without shock | ICD-10 | O85 | Puerperal sepsis |
| Sepsis without shock | ICD-10 | R65 | Symptoms and signs specifically associated with systemic inflammation and infection |
| Sepsis without shock | ICD-10 | R65.1 | Systemic inflammatory response syndrome (SIRS) of non-infectious origin |
| Sepsis without shock | ICD-10 | R65.10 | Systemic inflammatory response syndrome (SIRS) of non-infectious origin without acute organ dysfunction |
| Sepsis without shock | ICD-10 | R65.11 | Systemic inflammatory response syndrome (SIRS) of non-infectious origin with acute organ dysfunction |
| Sepsis without shock | ICD-10 | R65.20 | Severe sepsis without septic shock |
| Sepsis without shock | ICD-10 | R78.81 | Bacteremia |
| Sepsis without shock | ICD-10 | T81.44 | Sepsis following a procedure |
| Sepsis without shock | ICD-10 | T81.44XA | Sepsis following a procedure initial encounter |
| Sepsis without shock | ICD-10 | T81.44XD | Sepsis following a procedure subsequent encounter |
| Sepsis without shock | ICD-10 | T81.44XS | Sepsis following a procedure sequela |
| Shock | ICD-9 | 785.5 | Shock without mention of trauma |
| Shock | ICD-9 | 785.50 | Shock, unspecified |
| Shock | ICD-9 | 785.51 | Cardiogenic shock |
| Shock | ICD-9 | 785.52 | Septic shock |
| Shock | ICD-9 | 785.59 | Other shock without mention of trauma |
| Shock | ICD-9 | 998.0 | Postoperative shock not elsewhere classified |
| Shock | ICD-9 | 998.00 | Postoperative shock, unspecified |
| Shock | ICD-9 | 998.01 | Postoperative shock, cardiogenic |
| Shock | ICD-9 | 998.02 | Postoperative shock, septic |
| Shock | ICD-9 | 998.09 | Postoperative shock, other |
| Shock | ICD-10 | R57 | Shock, not elsewhere classified |
| Shock | ICD-10 | R57.0 | Cardiogenic shock |
| Shock | ICD-10 | R57.1 | Hypovolemic shock |
| Shock | ICD-10 | R57.8 | Other shock |
| Shock | ICD-10 | R57.9 | Shock, unspecified |
| Shock | ICD-10 | R65.21 | Severe sepsis with septic shock |
| Shock | ICD-10 | T81.1 | Postprocedural shock |
| Shock | ICD-10 | T81.10 | Postprocedural shock unspecified |
| Shock | ICD-10 | T81.10XA | Postprocedural shock, unspecified, initial encounter |
| Shock | ICD-10 | T81.10XD | Postprocedural shock, unspecified, subsequent encounter |
| Shock | ICD-10 | T81.10XS | Postprocedural shock, unspecified, sequela |
| Shock | ICD-10 | T81.11 | Postprocedural cardiogenic shock |
| Shock | ICD-10 | T81.11XA | Postprocedural cardiogenic shock, unspecified, initial encounter |
| Shock | ICD-10 | T81.11XD | Postprocedural cardiogenic shock, unspecified, subsequent encounter |
| Shock | ICD-10 | T81.11XS | Postprocedural cardiogenic shock, unspecified, sequela |
| Shock | ICD-10 | T81.12 | Postprocedural septic shock |
| Shock | ICD-10 | T81.12XA | Postprocedural septic shock, unspecified, initial encounter |
| Shock | ICD-10 | T81.12XD | Postprocedural septic shock, unspecified, subsequent encounter |
| Shock | ICD-10 | T81.12XS | Postprocedural septic shock, unspecified, sequela |
| Shock | ICD-10 | T81.19 | Other postprocedural shock |
| Shock | ICD-10 | T81.19XA | Other postprocedural shock, unspecified, initial encounter |
| Shock | ICD-10 | T81.19XD | Other postprocedural shock, unspecified, subsequent encounter |
| Shock | ICD-10 | T81.19XS | Other postprocedural shock, unspecified, sequela |
| Sickle cell | ICD-9 | 282.5 | Sickle cell trait |
| Sickle cell | ICD-9 | 282.6 | Sickle cell anemia, unspecified |
| Sickle cell | ICD-9 | 282.61 | Sickle cell anemia, without crisis |
| Sickle cell | ICD-9 | 282.62 | Sickle cell anemia, with crisis |
| Sickle cell | ICD-9 | 282.63 | Sickle cell/Hb-C disease without crisis |
| Sickle cell | ICD-9 | 282.64 | Sickle cell/Hb-C disease with crisis |
| Sickle cell | ICD-9 | 282.68 | Other sickle cell disease without crisis |
| Sickle cell | ICD-9 | 282.69 | Other sickle cell disease with crisis |
| Sickle cell | ICD-10 | D57.0 | Sickle-cell anemia with crisis |
| Sickle cell | ICD-10 | D57.00 | Hb-SS disease with crisis, unspecified |
| Sickle cell | ICD-10 | D57.01 | Hb-SS disease with acute chest syndrome |
| Sickle cell | ICD-10 | D57.02 | Hb-SS disease with splenic sequestration |
| Sickle cell | ICD-10 | D57.03 | Hb-SS disease with cerebral vascular involvement |
| Sickle cell | ICD-10 | D57.09 | Hb-SS disease with crisis with other specified complication |
| Sickle cell | ICD-10 | D57.1 | Sickle-cell anemia without crisis |
| Sickle cell | ICD-10 | D57.2 | Double heterozygous sickling disorders |
| Sickle cell | ICD-10 | D57.20 | Sickle-cell/Hb-C disease without crisis |
| Sickle cell | ICD-10 | D57.21 | Sickle-cell/Hb-C disease with crisis |
| Sickle cell | ICD-10 | D57.211 | Sickle-cell/Hb-C disease with acute chest syndrome |
| Sickle cell | ICD-10 | D57.212 | Sickle-cell/Hb-C disease with splenic sequestration |
| Sickle cell | ICD-10 | D57.213 | Sickle-cell/Hb-C disease with cerebral vascular involvement |
| Sickle cell | ICD-10 | D57.218 | Sickle-cell/Hb-C disease with crisis with other specified complication |
| Sickle cell | ICD-10 | D57.219 | Sickle-cell/Hb-C disease with crisis, unspecified |
| Sickle cell | ICD-10 | D57.3 | Sickle-cell trait |
| Sickle cell | ICD-10 | D57.8 | Other sickle-cell disorders |
| Sickle cell | ICD-10 | D57.80 | Other sickle-cell disorders without crisis |
| Sickle cell | ICD-10 | D57.81 | Other sickle-cell disorders with crisis |
| Sickle cell | ICD-10 | D57.811 | Other sickle-cell disorders with acute chest syndrome |
| Sickle cell | ICD-10 | D57.812 | Other sickle-cell disorders with splenic sequestration |
| Sickle cell | ICD-10 | D57.813 | Other sickle-cell disorders with cerebral vascular involvement |
| Sickle cell | ICD-10 | D57.818 | Other sickle-cell disorders with crisis with other specified complication |
| Sickle cell | ICD-10 | D57.819 | Other sickle-cell disorders with crisis, unspecified |
| Thalassemia | ICD-9 | 282.4 | Thalassemias |
| Thalassemia | ICD-9 | 282.40 | Thalassemia, unspecified |
| Thalassemia | ICD-9 | 282.41 | Sickle-cell thalassemia without crisis |
| Thalassemia | ICD-9 | 282.42 | Sickle-cell thalassemia with crisis |
| Thalassemia | ICD-9 | 282.43 | Alpha thalassemia |
| Thalassemia | ICD-9 | 282.44 | Beta thalassemia |
| Thalassemia | ICD-9 | 282.45 | Delta-beta thalassemia |
| Thalassemia | ICD-9 | 282.46 | Thalassemia minor |
| Thalassemia | ICD-9 | 282.47 | Hemoglobin E-beta thalassemia |
| Thalassemia | ICD-9 | 282.49 | Other thalassemia |
| Thalassemia | ICD-10 | D56 | Thalassemia |
| Thalassemia | ICD-10 | D56.0 | Alpha thalassemia |
| Thalassemia | ICD-10 | D56.1 | Beta thalassemia |
| Thalassemia | ICD-10 | D56.2 | Delta-beta thalassemia |
| Thalassemia | ICD-10 | D56.3 | Thalassemia minor |
| Thalassemia | ICD-10 | D56.4 | Hereditary persistence of fetal hemoglobin [HPFH] |
| Thalassemia | ICD-10 | D56.5 | Hemoglobin E-beta thalassemia |
| Thalassemia | ICD-10 | D56.8 | Other thalassemias |
| Thalassemia | ICD-10 | D56.9 | Thalassemia, unspecified |
| Thyrotoxicosis | ICD-9 | 242 | Thyrotoxicosis with or without goiter |
| Thyrotoxicosis | ICD-9 | 242.0 | Toxic diffuse goiter |
| Thyrotoxicosis | ICD-9 | 242.00 | Toxic diffuse goiter without mention of thyrotoxic crisis or storm |
| Thyrotoxicosis | ICD-9 | 242.01 | Toxic diffuse goiter with mention of thyrotoxic crisis or storm |
| Thyrotoxicosis | ICD-9 | 242.1 | Toxic uninodular goiter |
| Thyrotoxicosis | ICD-9 | 242.10 | Toxic uninodular goiter without mention of thyrotoxic crisis or storm |
| Thyrotoxicosis | ICD-9 | 242.11 | Toxic uninodular goiter with mention of thyrotoxic crisis or storm |
| Thyrotoxicosis | ICD-9 | 242.2 | Toxic multinodular goiter |
| Thyrotoxicosis | ICD-9 | 242.20 | Toxic multinodular goiter without mention of thyrotoxic crisis or storm |
| Thyrotoxicosis | ICD-9 | 242.21 | Toxic multinodular goiter with mention of thyrotoxic crisis or storm |
| Thyrotoxicosis | ICD-9 | 242.3 | Toxic nodular goiter, unspecified |
| Thyrotoxicosis | ICD-9 | 242.30 | Toxic nodular goiter, unspecified type, without mention of thyrotoxic crisis or storm |
| Thyrotoxicosis | ICD-9 | 242.31 | Toxic nodular goiter, unspecified type, with mention of thyrotoxic crisis or storm |
| Thyrotoxicosis | ICD-9 | 242.4 | Thyrotoxicosis from ectopic thyroid nodule |
| Thyrotoxicosis | ICD-9 | 242.40 | Thyrotoxicosis from ectopic thyroid nodule without mention of thyrotoxic crisis or storm |
| Thyrotoxicosis | ICD-9 | 242.41 | Thyrotoxicosis from ectopic thyroid nodule with mention of thyrotoxic crisis or storm |
| Thyrotoxicosis | ICD-9 | 242.8 | Thyrotoxicosis from other specified origin |
| Thyrotoxicosis | ICD-9 | 242.80 | Thyrotoxicosis of other specified origin without mention of thyrotoxic crisis or storm |
| Thyrotoxicosis | ICD-9 | 242.81 | Thyrotoxicosis of other specified origin with mention of thyrotoxic crisis or storm |
| Thyrotoxicosis | ICD-9 | 242.9 | Thyrotoxicosis without mention of goiter or other cause, and without mention of thyrotoxic crisis or storm |
| Thyrotoxicosis | ICD-9 | 242.90 | Thyrotoxicosis without mention of goiter or other cause, and without mention of thyrotoxic crisis or storm |
| Thyrotoxicosis | ICD-9 | 242.91 | Thyrotoxicosis without mention of goiter or other cause, with mention of thyrotoxic crisis or storm |
| Thyrotoxicosis | ICD-10 | E05 | Thyrotoxicosis [hyperthyroidism] |
| Thyrotoxicosis | ICD-10 | E05.0 | Thyrotoxicosis with diffuse goiter |
| Thyrotoxicosis | ICD-10 | E05.00 | Thyrotoxicosis with diffuse goiter without thyrotoxic crisis or storm |
| Thyrotoxicosis | ICD-10 | E05.01 | Thyrotoxicosis with diffuse goiter with thyrotoxic crisis or storm |
| Thyrotoxicosis | ICD-10 | E05.1 | Thyrotoxicosis with toxic single thyroid nodule |
| Thyrotoxicosis | ICD-10 | E05.10 | Thyrotoxicosis with toxic single thyroid nodule without thyrotoxic crisis or storm |
| Thyrotoxicosis | ICD-10 | E05.11 | Thyrotoxicosis with toxic single thyroid nodule with thyrotoxic crisis or storm |
| Thyrotoxicosis | ICD-10 | E05.2 | Thyrotoxicosis with toxic multinodular goiter |
| Thyrotoxicosis | ICD-10 | E05.20 | Thyrotoxicosis with toxic multinodular goiter without thyrotoxic crisis or storm |
| Thyrotoxicosis | ICD-10 | E05.21 | Thyrotoxicosis with toxic multinodular goiter with thyrotoxic crisis or storm |
| Thyrotoxicosis | ICD-10 | E05.3 | Thyrotoxicosis from ectopic thyroid tissue |
| Thyrotoxicosis | ICD-10 | E05.30 | Thyrotoxicosis from ectopic thyroid tissue without thyrotoxic crisis or storm |
| Thyrotoxicosis | ICD-10 | E05.31 | Thyrotoxicosis from ectopic thyroid tissue with thyrotoxic crisis or storm |
| Thyrotoxicosis | ICD-10 | E05.4 | Thyrotoxicosis factitia |
| Thyrotoxicosis | ICD-10 | E05.40 | Thyrotoxicosis factitia without thyrotoxic crisis or storm |
| Thyrotoxicosis | ICD-10 | E05.41 | Thyrotoxicosis factitia with thyrotoxic crisis or storm |
| Thyrotoxicosis | ICD-10 | E05.5 | Thyroid crisis or storm |
| Thyrotoxicosis | ICD-10 | E05.8 | Other thyrotoxicosis |
| Thyrotoxicosis | ICD-10 | E05.80 | Other thyrotoxicosis without thyrotoxic crisis or storm |
| Thyrotoxicosis | ICD-10 | E05.81 | Other thyrotoxicosis with thyrotoxic crisis or storm |
| Thyrotoxicosis | ICD-10 | E05.9 | Thyrotoxicosis, unspecified |
| Thyrotoxicosis | ICD-10 | E05.90 | Thyrotoxicosis, unspecified without thyrotoxic crisis or storm |
| Thyrotoxicosis | ICD-10 | E05.91 | Thyrotoxicosis, unspecified with thyrotoxic crisis or storm |

Abbreviations: ICD-9=International Classification of Diseases, Ninth Revision; ICD-10=International Classification of Diseases, Tenth Revision

# **Table S2**. List of International Classification of Diseases, Ninth Revision (ICD-9) and International Classification of Diseases, Tenth Revision (ICD-10) diagnostic codes used to identify chronic liver disease.

| **Type of Chronic Liver Disease** | **Code Type** | **Code** | **Description** |
| --- | --- | --- | --- |
| Alcohol dependence | ICD-9 | 291.1 | Alcohol-induced persisting amnestic disorder |
| Alcohol dependence | ICD-9 | 291.2 | Alcohol-induced persisting dementia |
| Alcohol dependence | ICD-9 | 291.3 | Alcohol-induced psychotic disorder with hallucinations |
| Alcohol dependence | ICD-9 | 291.5 | Alcohol-induced psychotic disorder with delusions |
| Alcohol dependence | ICD-9 | 291.82 | Alcohol induced sleep disorders |
| Alcohol dependence | ICD-9 | 291.89 | Other alcohol-induced mental disorders |
| Alcohol dependence | ICD-9 | 291.9 | Unspecified alcohol-induced mental disorders |
| Alcohol dependence | ICD-9 | 303.00 | Acute alcoholic intoxication in alcoholism, unspecified |
| Alcohol dependence | ICD-9 | 303.01 | Acute alcoholic intoxication in alcoholism, continuous |
| Alcohol dependence | ICD-9 | 303.02 | Acute alcoholic intoxication in alcoholism, episodic |
| Alcohol dependence | ICD-9 | 303.90 | Other and unspecified alcohol dependence, unspecified |
| Alcohol dependence | ICD-9 | 303.91 | Other and unspecified alcohol dependence, continuous |
| Alcohol dependence | ICD-9 | 303.92 | Other and unspecified alcohol dependence, episodic |
| Alcohol dependence | ICD-10 | F10.20 | Alcohol dependence, uncomplicated |
| Alcohol dependence | ICD-10 | F10.22 | Alcohol dependence with intoxication |
| Alcohol dependence | ICD-10 | F10.220 | Alcohol dependence with intoxication, uncomplicated |
| Alcohol dependence | ICD-10 | F10.221 | Alcohol dependence with intoxication delirium |
| Alcohol dependence | ICD-10 | F10.229 | Alcohol dependence with intoxication, unspecified |
| Alcohol dependence | ICD-10 | F10.24 | Alcohol dependence with alcohol-induced mood disorder |
| Alcohol dependence | ICD-10 | F10.25 | Alcohol dependence with alcohol-induced psychotic disorder |
| Alcohol dependence | ICD-10 | F10.250 | Alcohol dependence with alcohol-induced psychotic disorder with delusions |
| Alcohol dependence | ICD-10 | F10.251 | Alcohol dependence with alcohol-induced psychotic disorder with hallucinations |
| Alcohol dependence | ICD-10 | F10.259 | Alcohol dependence with alcohol-induced psychotic disorder, unspecified |
| Alcohol dependence | ICD-10 | F10.26 | Alcohol dependence with alcohol-induced persisting amnestic disorder |
| Alcohol dependence | ICD-10 | F10.27 | Alcohol dependence with alcohol-induced persisting dementia |
| Alcohol dependence | ICD-10 | F10.28 | Alcohol dependence with other alcohol-induced disorders |
| Alcohol dependence | ICD-10 | F10.280 | Alcohol dependence with alcohol-induced anxiety disorder |
| Alcohol dependence | ICD-10 | F10.281 | Alcohol dependence with alcohol-induced sexual dysfunction |
| Alcohol dependence | ICD-10 | F10.282 | Alcohol dependence with alcohol-induced sleep disorder |
| Alcohol dependence | ICD-10 | F10.288 | Alcohol dependence with other alcohol-induced disorder |
| Alcohol dependence | ICD-10 | F10.29 | Alcohol dependence with unspecified alcohol-induced disorder |
| Alcohol withdrawal with abuse/dependence | ICD-9 | 291.0 | Alcohol withdrawal delirium |
| Alcohol withdrawal with abuse/dependence | ICD-9 | 291.81 | Alcohol withdrawal |
| Alcohol withdrawal with abuse/dependence | ICD-10 | F10.13 | Alcohol abuse with withdrawal |
| Alcohol withdrawal with abuse/dependence | ICD-10 | F10.130 | Alcohol abuse with withdrawal, uncomplicated |
| Alcohol withdrawal with abuse/dependence | ICD-10 | F10.131 | Alcohol abuse with withdrawal delirium |
| Alcohol withdrawal with abuse/dependence | ICD-10 | F10.132 | Alcohol abuse with withdrawal with perceptual disturbance |
| Alcohol withdrawal with abuse/dependence | ICD-10 | F10.139 | Alcohol abuse with withdrawal, unspecified |
| Alcohol withdrawal with abuse/dependence | ICD-10 | F10.23 | Alcohol dependence with withdrawal |
| Alcohol withdrawal with abuse/dependence | ICD-10 | F10.230 | Alcohol dependence with withdrawal, uncomplicated |
| Alcohol withdrawal with abuse/dependence | ICD-10 | F10.231 | Alcohol dependence with withdrawal delirium |
| Alcohol withdrawal with abuse/dependence | ICD-10 | F10.232 | Alcohol dependence with withdrawal with perceptual disturbance |
| Alcohol withdrawal with abuse/dependence | ICD-10 | F10.239 | Alcohol dependence with withdrawal, unspecified |
| Alcohol withdrawal with abuse/dependence | ICD-10 | F10.93 | Alcohol use, unspecified with withdrawal |
| Alcohol withdrawal with abuse/dependence | ICD-10 | F10.930 | Alcohol use, unspecified with withdrawal, uncomplicated |
| Alcohol withdrawal with abuse/dependence | ICD-10 | F10.931 | Alcohol use, unspecified with withdrawal delirium |
| Alcohol withdrawal with abuse/dependence | ICD-10 | F10.932 | Alcohol use, unspecified with withdrawal with perceptual disturbance |
| Alcohol withdrawal with abuse/dependence | ICD-10 | F10.939 | Alcohol use, unspecified with withdrawal, unspecified |
| Alcoholic liver disease | ICD-9 | 571.0 | Alcoholic fatty liver |
| Alcoholic liver disease | ICD-9 | 571.1 | Acute alcoholic hepatitis |
| Alcoholic liver disease | ICD-9 | 571.2 | Alcoholic cirrhosis of the liver |
| Alcoholic liver disease | ICD-9 | 571.3 | Alcoholic liver damage, unspecified |
| Alcoholic liver disease | ICD-10 | K70 | Alcoholic liver disease |
| Alcoholic liver disease | ICD-10 | K70.0 | Alcoholic fatty liver |
| Alcoholic liver disease | ICD-10 | K70.1 | Alcoholic hepatitis |
| Alcoholic liver disease | ICD-10 | K70.10 | Alcoholic hepatitis without ascites |
| Alcoholic liver disease | ICD-10 | K70.11 | Alcoholic hepatitis with ascites |
| Alcoholic liver disease | ICD-10 | K70.2 | Alcoholic fibrosis/sclerosis of the liver |
| Alcoholic liver disease | ICD-10 | K70.3 | Alcoholic cirrhosis of liver |
| Alcoholic liver disease | ICD-10 | K70.30 | Alcoholic cirrhosis of liver without ascites |
| Alcoholic liver disease | ICD-10 | K70.31 | Alcoholic cirrhosis of liver with ascites |
| Alcoholic liver disease | ICD-10 | K70.4 | Alcoholic hepatic failure |
| Alcoholic liver disease | ICD-10 | K70.40 | Alcoholic hepatic failure without coma |
| Alcoholic liver disease | ICD-10 | K70.41 | Alcoholic hepatic failure with coma |
| Alcoholic liver disease | ICD-10 | K70.9 | Alcoholic liver disease, unspecified |
| Alpha-1-antitrypsin deficiency | ICD-9 | 273.4 | Alpha-1-antitrypsin deficiency |
| Alpha-1-antitrypsin deficiency | ICD-10 | E88.01 | Alpha-1-antitrypsin deficiency |
| Autoimmune hepatitis | ICD-9 | 571.42 | Autoimmune hepatitis |
| Autoimmune hepatitis | ICD-10 | K75.4 | Autoimmune hepatitis |
| Cirrhosis (compensated or decompensated) | ICD-9 | 456.0 | Esophageal varices with bleeding |
| Cirrhosis (compensated or decompensated) | ICD-9 | 456.1 | Esophageal varices without bleeding |
| Cirrhosis (compensated or decompensated) | ICD-9 | 456.2 | Esophageal varices in diseases classified elsewhere |
| Cirrhosis (compensated or decompensated) | ICD-9 | 456.20 | Esophageal varices in diseases classified elsewhere with bleeding |
| Cirrhosis (compensated or decompensated) | ICD-9 | 456.21 | Esophageal varices in diseases classified elsewhere without bleeding |
| Cirrhosis (compensated or decompensated) | ICD-9 | 567.23 | Spontaneous bacterial peritonitis |
| Cirrhosis (compensated or decompensated) | ICD-9 | 571.5 | Cirrhosis of liver without mention of alcohol |
| Cirrhosis (compensated or decompensated) | ICD-9 | 572.2 | Hepatic encephalopathy |
| Cirrhosis (compensated or decompensated) | ICD-9 | 572.3 | Portal hypertension |
| Cirrhosis (compensated or decompensated) | ICD-9 | 572.4 | Hepatorenal syndrome |
| Cirrhosis (compensated or decompensated) | ICD-9 | 789.5 | Ascites |
| Cirrhosis (compensated or decompensated) | ICD-9 | 789.59 | Other ascites |
| Cirrhosis (compensated or decompensated) | ICD-10 | I85 | Esophageal varices |
| Cirrhosis (compensated or decompensated) | ICD-10 | I85.0 | Esophageal varices |
| Cirrhosis (compensated or decompensated) | ICD-10 | I85.00 | Esophageal varices without bleeding |
| Cirrhosis (compensated or decompensated) | ICD-10 | I85.01 | Esophageal varices with bleeding |
| Cirrhosis (compensated or decompensated) | ICD-10 | I85.1 | Secondary esophageal varices |
| Cirrhosis (compensated or decompensated) | ICD-10 | I85.10 | Secondary esophageal varices without bleeding |
| Cirrhosis (compensated or decompensated) | ICD-10 | I85.11 | Secondary esophageal varices with bleeding |
| Cirrhosis (compensated or decompensated) | ICD-10 | K65.2 | Spontaneous bacterial peritonitis |
| Cirrhosis (compensated or decompensated) | ICD-10 | K71.51 | Toxic liver disease with chronic active hepatitis with ascites |
| Cirrhosis (compensated or decompensated) | ICD-10 | K72.1 | Chronic hepatic failure |
| Cirrhosis (compensated or decompensated) | ICD-10 | K72.10 | Chronic hepatic failure without coma |
| Cirrhosis (compensated or decompensated) | ICD-10 | K72.11 | Chronic hepatic failure with coma |
| Cirrhosis (compensated or decompensated) | ICD-10 | K72.9 | Hepatic failure, unspecified |
| Cirrhosis (compensated or decompensated) | ICD-10 | K72.90 | Hepatic failure, unspecified without coma |
| Cirrhosis (compensated or decompensated) | ICD-10 | K72.91 | Hepatic failure, unspecified with coma |
| Cirrhosis (compensated or decompensated) | ICD-10 | K74.1 | Hepatic sclerosis |
| Cirrhosis (compensated or decompensated) | ICD-10 | K74.2 | Hepatic fibrosis with hepatic sclerosis |
| Cirrhosis (compensated or decompensated) | ICD-10 | K74.60 | Unspecified cirrhosis of liver |
| Cirrhosis (compensated or decompensated) | ICD-10 | K76.7 | Hepatorenal syndrome |
| Cirrhosis (compensated or decompensated) | ICD-10 | K76.81 | Hepatopulmonary syndrome |
| Cirrhosis (compensated or decompensated) | ICD-10 | R18.8 | Other ascites |
| Congestive hepatopathy | ICD-9 | 573.0 | Chronic passive congestion of the liver |
| Congestive hepatopathy | ICD-10 | K76.1 | Chronic passive congestion of the liver |
| Hemochromatosis | ICD-9 | 275.01 | Hereditary hemochromatosis |
| Hemochromatosis | ICD-9 | 275.02 | Hemochromatosis due to repeated red blood cell transfusions |
| Hemochromatosis | ICD-9 | 275.03 | Other hemochromatosis |
| Hemochromatosis | ICD-10 | E83.110 | Hereditary hemochromatosis |
| Hepatitis B | ICD-9 | 070.2 | Viral hepatitis B with hepatic coma |
| Hepatitis B | ICD-9 | 070.20 | Viral hepatitis B with hepatic coma, acute or unspecified, without mention of hepatitis delta |
| Hepatitis B | ICD-9 | 070.22 | Chronic viral hepatitis B with hepatic coma without mention of hepatitis delta |
| Hepatitis B | ICD-9 | 070.3 | Viral hepatitis B without mention of hepatic coma |
| Hepatitis B | ICD-9 | 070.30 | Viral hepatitis B without mention of hepatic coma, acute or unspecified, without mention of hepatitis delta |
| Hepatitis B | ICD-9 | 070.32 | Chronic viral hepatitis B without mention of hepatic coma without mention of hepatitis delta |
| Hepatitis B | ICD-9 | V02.61 | Hepatitis B Carrier |
| Hepatitis B | ICD-10 | B18.1 | Chronic viral hepatitis B without delta-agent |
| Hepatitis B | ICD-10 | B19.1 | Unspecified viral hepatitis B |
| Hepatitis B | ICD-10 | B19.10 | Unspecified viral hepatitis B without hepatic coma |
| Hepatitis B | ICD-10 | B19.11 | Unspecified viral hepatitis B with hepatic coma |
| Hepatitis B and D | ICD-9 | 070.21 | Viral hepatitis B with hepatic coma, acute or unspecified, with hepatitis delta |
| Hepatitis B and D | ICD-9 | 070.23 | Chronic viral hepatitis B with hepatic coma with hepatitis delta |
| Hepatitis B and D | ICD-9 | 070.31 | Viral hepatitis B without mention of hepatic coma, acute or unspecified, with hepatitis delta |
| Hepatitis B and D | ICD-9 | 070.33 | Chronic viral hepatitis B without mention of hepatic coma with mention of hepatitis delta |
| Hepatitis B and D | ICD-9 | 070.42 | Hepatitis delta without mention of active hepatitis B disease with hepatic coma |
| Hepatitis B and D | ICD-9 | 070.52 | Hepatitis delta without mention of active hepatitis B disease or hepatic coma |
| Hepatitis B and D | ICD-10 | B18.0 | Chronic viral hepatitis B with delta-agent |
| Hepatitis C | ICD-9 | 070.44 | Chronic hepatitis C with hepatic coma |
| Hepatitis C | ICD-9 | 070.54 | Chronic hepatitis C without mention of hepatic coma |
| Hepatitis C | ICD-9 | 070.7 | Unspecified viral hepatitis C |
| Hepatitis C | ICD-9 | 070.70 | Unspecified viral hepatitis C without hepatic coma |
| Hepatitis C | ICD-9 | 070.71 | Unspecified viral hepatitis C with hepatic coma |
| Hepatitis C | ICD-9 | V02.62 | Hepatitis C carrier |
| Hepatitis C | ICD-10 | B18.2 | Chronic viral hepatitis C |
| Hepatitis C | ICD-10 | B19.2 | Unspecified viral hepatitis C |
| Hepatitis C | ICD-10 | B19.20 | Unspecified viral hepatitis C without hepatic coma |
| Hepatitis C | ICD-10 | B19.21 | Unspecified viral hepatitis C with hepatic coma |
| Non-alcoholic fatty liver disease | ICD-9 | 571.8 | Other chronic non-alcoholic liver disease |
| Non-alcoholic fatty liver disease | ICD-9 | 571.9 | Unspecified chronic liver disease without mention of alcohol |
| Non-alcoholic fatty liver disease | ICD-10 | K76.0 | Fatty change of liver, not elsewhere classified |
| Other acute or unspecified viral hepatitis | ICD-10 | B18 | Chronic viral hepatitis |
| Other acute or unspecified viral hepatitis | ICD-10 | B18.8 | Other chronic viral hepatitis |
| Other acute or unspecified viral hepatitis | ICD-10 | B18.9 | Chronic viral hepatitis, unspecified |
| Other unspecified hepatitis/liver disease | ICD-9 | 571 | Chronic liver disease and cirrhosis |
| Other unspecified hepatitis/liver disease | ICD-9 | 571.4 | Chronic hepatitis |
| Other unspecified hepatitis/liver disease | ICD-9 | 571.40 | Chronic hepatitis, unspecified |
| Other unspecified hepatitis/liver disease | ICD-9 | 571.41 | Chronic persistent hepatitis |
| Other unspecified hepatitis/liver disease | ICD-9 | 571.49 | Other chronic hepatitis |
| Other unspecified hepatitis/liver disease | ICD-10 | K73 | Chronic hepatitis, not elsewhere classified |
| Other unspecified hepatitis/liver disease | ICD-10 | K73.0 | Chronic persistent hepatitis, not elsewhere classified |
| Other unspecified hepatitis/liver disease | ICD-10 | K73.1 | Chronic lobular hepatitis, not elsewhere classified |
| Other unspecified hepatitis/liver disease | ICD-10 | K73.2 | Chronic active hepatitis, not elsewhere classified |
| Other unspecified hepatitis/liver disease | ICD-10 | K73.8 | Other chronic hepatitis, not elsewhere classified |
| Other unspecified hepatitis/liver disease | ICD-10 | K73.9 | Chronic hepatitis, unspecified |
| Primary/secondary biliary cirrhosis | ICD-9 | 571.6 | Biliary cirrhosis |
| Primary/secondary biliary cirrhosis | ICD-10 | K74.3 | Primary biliary cirrhosis |
| Primary/secondary biliary cirrhosis | ICD-10 | K74.4 | Secondary biliary cirrhosis |
| Primary/secondary biliary cirrhosis | ICD-10 | K74.5 | Biliary cirrhosis, unspecified |
| Wilson's disease | ICD-9 | 275.1 | Disorders of copper metabolism |
| Wilson's disease | ICD-10 | E83.01 | Wilson's disease |

Abbreviations: ICD-9=International Classification of Diseases, Ninth Revision; ICD-10=International Classification of Diseases, Tenth Revision

# **Table S3**. Drugs with p<0.3 in Days 1-133 excluding all drugs with an Anatomical Therapeutic Chemical (ATC) code starting with “A” (i.e., alimentary tract and metabolism) except for “A02” (i.e., drugs for acid related disorders) among people without chronic liver disease.

| **Node** | **Drug Description** | **Total Node Cases** | **Observed Cases in Window** | **Expected Cases in Window** | **Observed**  **/Expected** | **Observed-Expected** | **Test Statistic** | ***P* Value** |
| --- | --- | --- | --- | --- | --- | --- | --- | --- |
| A02BA02 | RANITIDINE | 727 | 354 | 288.87 | 1.23 | 66.35 | 12.17 | 0.001 |
| A02BC01 | OMEPRAZOLE | 1190 | 551 | 472.84 | 1.17 | 80.58 | 10.89 | 0.001 |
| J01CR02 | AMOXICILLIN AND BETA-LACTAMASE INHIBITOR | 489 | 242 | 194.30 | 1.25 | 48.29 | 9.64 | 0.002 |
| J01MA02 | CIPROFLOXACIN | 698 | 332 | 277.34 | 1.20 | 55.63 | 8.94 | 0.004 |
| B01AB01 | HEPARIN | 32 | 24 | 12.71 | 1.89 | 11.29 | 8.21 | 0.017 |
| R01AX06 | MUPIROCIN | 21 | 17 | 8.34 | 2.04 | 8.66 | 7.49 | 0.025 |
| R06AD02 | PROMETHAZINE | 172 | 93 | 68.34 | 1.36 | 24.76 | 7.22 | 0.040 |
| N05AB04 | PROCHLORPERAZINE | 114 | 63 | 45.30 | 1.39 | 17.75 | 5.60 | 0.125 |
| J04AK02 | ETHAMBUTOL | 6 | 6 | 2.38 | 2.52 | 3.62 | 5.54 | 0.242 |
| L01EX03 | PAZOPANIB | 6 | 6 | 2.38 | 2.52 | 3.62 | 5.54 | 0.242 |

# **Table S4**. Drugs with p<0.3 in Days 1-84 among people without chronic liver disease.

| **Node** | **Drug Description** | **Total Node Cases** | **Observed Cases in Window** | **Expected Cases in Window** | **Observed**  **/Expected** | **Observed-Expected** | **Test Statistic** | ***P* Value** |
| --- | --- | --- | --- | --- | --- | --- | --- | --- |
| A04AA01 | ONDANSETRON | 286 | 132 | 77.29 | 1.72 | 55.05 | 24.00 | 0.001 |
| A02BA02 | RANITIDINE | 727 | 268 | 196.48 | 1.37 | 72.68 | 17.02 | 0.001 |
| R01AX06 | MUPIROCIN | 21 | 17 | 5.68 | 3.00 | 11.33 | 13.28 | 0.001 |
| J01CR02 | AMOXICILLIN AND BETA-LACTAMASE INHIBITOR | 489 | 178 | 132.16 | 1.35 | 46.34 | 10.36 | 0.002 |
| R06AD02 | PROMETHAZINE | 172 | 74 | 46.48 | 1.60 | 27.62 | 10.20 | 0.002 |
| A03AA07 | DICYCLOMINE | 103 | 49 | 27.84 | 1.76 | 21.21 | 9.88 | 0.004 |
| A02BC01 | OMEPRAZOLE | 1190 | 389 | 321.61 | 1.22 | 69.20 | 9.56 | 0.004 |
| N02AJ22 | HYDROCODONE/ACETAMINOPHEN | 1141 | 373 | 308.37 | 1.22 | 66.30 | 9.16 | 0.005 |
| J01MA02 | CIPROFLOXACIN | 698 | 236 | 188.64 | 1.26 | 48.10 | 7.90 | 0.024 |
| L01EX03 | PAZOPANIB | 6 | 6 | 1.62 | 3.70 | 4.38 | 7.85 | 0.036 |
| B01AB01 | HEPARIN | 32 | 19 | 8.65 | 2.20 | 10.36 | 7.35 | 0.042 |
| N05AB04 | PROCHLORPERAZINE | 114 | 49 | 30.81 | 1.59 | 18.24 | 6.72 | 0.061 |
| A02AF02 | ORDINARY SALT COMBINATIONS AND ANTIFLATULENTS | 122 | 51 | 32.97 | 1.55 | 18.08 | 6.20 | 0.130 |
| A06AA02 | DOCUSATE | 619 | 206 | 167.29 | 1.24 | 39.24 | 5.96 | 0.161 |
| C01BB01 | LIDOCAINE | 7 | 6 | 1.89 | 3.17 | 4.11 | 5.30 | 0.240 |
| A15zzzz | APPETITE STIMULANTS | 39 | 20 | 10.54 | 1.90 | 9.47 | 5.14 | 0.266 |

# **Table S5**. Drugs with p<0.3 in Days 1-28 among people without chronic liver disease.

| **Node** | **Drug Description** | **Total Node Cases** | **Observed Cases in Window** | **Expected Cases in Window** | **Observed**  **/Expected** | **Observed-Expected** | **Test Statistic** | ***P* Value** |
| --- | --- | --- | --- | --- | --- | --- | --- | --- |
| A04AA01 | ONDANSETRON | 286 | 73 | 29.45 | 2.50 | 43.82 | 26.86 | 0.001 |
| A02BA02 | RANITIDINE | 727 | 132 | 74.87 | 1.79 | 58.06 | 20.69 | 0.001 |
| R01AX06 | MUPIROCIN | 21 | 13 | 2.16 | 6.02 | 10.84 | 16.48 | 0.001 |
| N02AJ22 | HYDROCODONE/ACETAMINOPHEN | 1141 | 178 | 117.51 | 1.54 | 62.05 | 15.70 | 0.001 |
| R06AD02 | PROMETHAZINE | 172 | 43 | 17.71 | 2.44 | 25.38 | 15.12 | 0.001 |
| J01MA02 | CIPROFLOXACIN | 698 | 108 | 71.89 | 1.51 | 36.68 | 9.07 | 0.005 |
| J01CR02 | AMOXICILLIN AND BETA-LACTAMASE INHIBITOR | 489 | 79 | 50.36 | 1.58 | 28.95 | 7.98 | 0.011 |
| J01MA16 | GATIFLOXACIN | 200 | 39 | 20.60 | 1.90 | 18.48 | 7.51 | 0.021 |
| A07BBzz | BISMUTH PREPARATIONS | 21 | 9 | 2.16 | 4.17 | 6.84 | 7.43 | 0.022 |
| A02BC01 | OMEPRAZOLE | 1190 | 164 | 122.56 | 1.35 | 42.56 | 7.36 | 0.025 |
| A06AA02 | DOCUSATE | 619 | 94 | 63.75 | 1.48 | 30.67 | 7.20 | 0.026 |
| A02AF02 | ORDINARY SALT COMBINATIONS AND ANTIFLATULENTS | 122 | 26 | 12.56 | 2.08 | 13.47 | 6.35 | 0.061 |
| A03AA07 | DICYCLOMINE | 103 | 23 | 10.61 | 2.17 | 12.42 | 6.30 | 0.066 |
| A03BA03 | HYOSCYAMINE | 17 | 7 | 1.75 | 4.00 | 5.25 | 5.48 | 0.152 |
| B01AB01 | HEPARIN | 32 | 10 | 3.30 | 3.04 | 6.71 | 5.25 | 0.198 |
| A15zzzz | APPETITE STIMULANTS | 39 | 11 | 4.02 | 2.74 | 6.99 | 4.85 | 0.276 |
| A06AD11 | LACTULOSE | 136 | 26 | 14.01 | 1.86 | 12.03 | 4.72 | 0.294 |

# **Table S6**. Drugs with p<0.3 in Days 1-133 among people without chronic liver disease in a sensitivity analysis increasing the minimum number of observed cases from 5 to 10.

| **Node** | **Drug Description** | **Total Node Cases** | **Observed Cases in Window** | **Expected Cases in Window** | **Observed**  **/Expected** | **Observed-Expected** | **Test Statistic** | ***P* Value** |
| --- | --- | --- | --- | --- | --- | --- | --- | --- |
| A04AA01 | ONDANSETRON | 286 | 167 | 115.20 | 1.45 | 52.12 | 19.13 | 0.001 |
| A02BA02 | RANITIDINE | 727 | 354 | 292.85 | 1.21 | 62.15 | 10.68 | 0.001 |
| A02BC01 | OMEPRAZOLE | 1190 | 551 | 479.35 | 1.15 | 73.58 | 9.09 | 0.004 |
| J01CR02 | AMOXICILLIN AND BETA-LACTAMASE INHIBITOR | 489 | 242 | 196.98 | 1.23 | 45.51 | 8.55 | 0.008 |
| B01AB01 | HEPARIN | 32 | 24 | 12.89 | 1.86 | 11.12 | 7.96 | 0.013 |
| J01MA02 | CIPROFLOXACIN | 698 | 332 | 281.16 | 1.18 | 51.63 | 7.70 | 0.020 |
| R01AX06 | MUPIROCIN | 21 | 17 | 8.46 | 2.01 | 8.54 | 7.30 | 0.030 |
| R06AD02 | PROMETHAZINE | 172 | 93 | 69.28 | 1.34 | 23.81 | 6.66 | 0.049 |
| A03AA07 | DICYCLOMINE | 103 | 58 | 41.49 | 1.40 | 16.55 | 5.38 | 0.150 |
| N05AB04 | PROCHLORPERAZINE | 114 | 63 | 45.92 | 1.37 | 17.12 | 5.20 | 0.170 |
| J04AC01 | ISONIAZID | 22 | 16 | 8.86 | 1.81 | 7.14 | 4.75 | 0.282 |

# **Table S7**. Drugs with p<0.3 in Days 1-133 excluding all drugs with an Anatomical Therapeutic Chemical code starting with “A” (i.e., alimentary tract and metabolism) except for “A02” (i.e., drugs for acid related disorders) among people with chronic liver disease.

| **Node** | **Drug Description** | **Total Node Cases** | **Observed Cases in Window** | **Expected Cases in Window** | **Observed**  **/Expected** | **Observed-Expected** | **Test Statistic** | ***P* Value** |
| --- | --- | --- | --- | --- | --- | --- | --- | --- |
| C03DA01 | SPIRONOLACTONE | 2260 | 1319 | 1027.96 | 1.30 | 301.22 | 77.88 | 0.001 |
| C03CA01 | FUROSEMIDE | 2629 | 1486 | 1195.80 | 1.26 | 302.07 | 66.90 | 0.001 |
| N02AA01 | MORPHINE | 652 | 418 | 296.56 | 1.42 | 122.63 | 46.10 | 0.001 |
| N02AA05 | OXYCODONE | 1198 | 685 | 544.91 | 1.26 | 142.64 | 33.47 | 0.001 |
| L01EX02 | SORAFENIB | 171 | 113 | 77.78 | 1.45 | 35.31 | 14.72 | 0.001 |
| J01MA02 | CIPROFLOXACIN | 1521 | 792 | 691.83 | 1.15 | 102.50 | 13.55 | 0.001 |
| R06AD02 | PROMETHAZINE | 483 | 275 | 219.69 | 1.25 | 55.71 | 12.80 | 0.001 |
| A02AF02 | ORDINARY SALT COMBINATIONS AND ANTIFLATULENTS | 189 | 120 | 85.97 | 1.40 | 34.13 | 12.39 | 0.001 |
| N02AB03 | FENTANYL | 121 | 82 | 55.04 | 1.49 | 27.01 | 12.22 | 0.001 |
| A02BC01 | OMEPRAZOLE | 2458 | 1236 | 1118.02 | 1.11 | 122.48 | 11.81 | 0.001 |
| A02BX02 | SUCRALFATE | 93 | 64 | 42.30 | 1.51 | 21.73 | 10.31 | 0.002 |
| P01AB01 | METRONIDAZOLE | 230 | 138 | 104.62 | 1.32 | 33.50 | 9.77 | 0.002 |
| N05AB04 | PROCHLORPERAZINE | 614 | 332 | 279.28 | 1.19 | 53.21 | 9.16 | 0.005 |
| V06DBzz | FAT/CARBOHYDRATES/PROTEINS/ MINERALS/VITAMINS~COMBINATIONS | 156 | 95 | 70.96 | 1.34 | 24.10 | 7.47 | 0.035 |
| B05AA01 | ALBUMIN | 30 | 23 | 13.65 | 1.69 | 9.36 | 6.07 | 0.137 |
| V06DXzz | OTHER COMBINATIONS OF NUTRIENTS | 56 | 38 | 25.47 | 1.49 | 12.54 | 5.70 | 0.174 |
| J01MA16 | GATIFLOXACIN | 280 | 155 | 127.36 | 1.22 | 27.76 | 5.50 | 0.250 |
| N05AA01 | CHLORPROMAZINE | 55 | 37 | 25.02 | 1.48 | 11.99 | 5.30 | 0.277 |

# **Table S8**. Drugs with p<0.3 in Days 1-84 among people with chronic liver disease.

| **Node** | **Drug Description** | **Total Node Cases** | **Observed Cases in Window** | **Expected Cases in Window** | **Observed**  **/Expected** | **Observed-Expected** | **Test Statistic** | ***P* Value** |
| --- | --- | --- | --- | --- | --- | --- | --- | --- |
| A06AD11 | LACTULOSE | 1951 | 916 | 654.27 | 1.41 | 268.13 | 76.824 | 0.001 |
| N02AA01 | MORPHINE | 652 | 350 | 218.65 | 1.61 | 132.41 | 56.082 | 0.001 |
| C03DA01 | SPIRONOLACTONE | 2260 | 996 | 757.89 | 1.33 | 244.87 | 55.563 | 0.001 |
| A06AB06 | SENNA GLYCOSIDES | 792 | 398 | 265.60 | 1.51 | 133.70 | 47.317 | 0.001 |
| N02AA05 | OXYCODONE | 1198 | 561 | 401.75 | 1.40 | 161.62 | 45.877 | 0.001 |
| C03CA01 | FUROSEMIDE | 2629 | 1107 | 881.64 | 1.27 | 232.85 | 43.264 | 0.001 |
| A04AA01 | ONDANSETRON | 1200 | 545 | 402.42 | 1.36 | 144.70 | 36.858 | 0.001 |
| A15zzzz | APPETITE STIMULANTS | 213 | 129 | 71.43 | 1.81 | 57.72 | 32.496 | 0.001 |
| A03FA01 | METOCLOPRAMIDE | 229 | 125 | 76.80 | 1.63 | 48.34 | 21.353 | 0.001 |
| A06AB56 | SENNA GLYCOSIDES~COMBINATIONS | 396 | 191 | 132.80 | 1.44 | 58.48 | 18.279 | 0.001 |
| R06AD02 | PROMETHAZINE | 483 | 223 | 161.97 | 1.38 | 61.39 | 16.585 | 0.001 |
| A06AA02 | DOCUSATE | 1250 | 513 | 419.19 | 1.23 | 95.27 | 15.544 | 0.001 |
| N02AB03 | FENTANYL | 121 | 69 | 40.58 | 1.70 | 28.46 | 13.979 | 0.001 |
| A12BA01 | POTASSIUM CHLORIDE | 1534 | 598 | 514.43 | 1.17 | 85.17 | 10.166 | 0.007 |
| A02AF02 | ORDINARY SALT COMBINATIONS AND ANTIFLATULENTS | 189 | 93 | 63.38 | 1.47 | 29.69 | 9.868 | 0.007 |
| J01MA02 | CIPROFLOXACIN | 1521 | 589 | 510.07 | 1.16 | 80.43 | 9.153 | 0.008 |
| A02BX02 | SUCRALFATE | 93 | 51 | 31.19 | 1.64 | 19.83 | 8.862 | 0.012 |
| A03AX13 | SILICONES | 424 | 183 | 142.19 | 1.29 | 41.02 | 8.520 | 0.015 |
| P01AB01 | METRONIDAZOLE | 230 | 107 | 77.13 | 1.39 | 29.95 | 8.308 | 0.015 |
| L01EX02 | SORAFENIB | 171 | 83 | 57.35 | 1.45 | 25.71 | 8.195 | 0.016 |
| A07AA02 | NYSTATIN | 205 | 96 | 68.75 | 1.40 | 27.32 | 7.751 | 0.024 |
| V06DBzz | FAT/CARBOHYDRATES/PROTEINS/ MINERALS/VITAMINS~COMBINATIONS | 156 | 76 | 52.31 | 1.45 | 23.73 | 7.651 | 0.030 |
| A02BC01 | OMEPRAZOLE | 2458 | 909 | 824.29 | 1.11 | 87.33 | 6.648 | 0.075 |
| A05AA02 | URSODIOL | 121 | 60 | 40.58 | 1.48 | 19.45 | 6.617 | 0.075 |
| A04AD10 | DRONABINOL | 48 | 28 | 16.10 | 1.74 | 11.91 | 6.165 | 0.143 |

# **Table S9**. Drugs with p<0.3 in Days 1-28 among people with chronic liver disease.

| **Node** | **Drug Description** | **Total Node Cases** | **Observed Cases in Window** | **Expected Cases in Window** | **Observed**  **/Expected** | **Observed-Expected** | **Test Statistic** | ***P* Value** |
| --- | --- | --- | --- | --- | --- | --- | --- | --- |
| N02AA01 | MORPHINE | 652 | 200 | 96.18 | 2.10 | 104.66 | 53.50 | 0.001 |
| A06AD11 | LACTULOSE | 1951 | 453 | 287.79 | 1.60 | 169.25 | 50.15 | 0.001 |
| A04AA01 | ONDANSETRON | 1200 | 307 | 177.01 | 1.75 | 131.92 | 48.52 | 0.001 |
| A06AB06 | SENNA GLYCOSIDES | 792 | 216 | 116.83 | 1.86 | 100.14 | 41.73 | 0.001 |
| C03DA01 | SPIRONOLACTONE | 2260 | 484 | 333.37 | 1.47 | 154.91 | 37.01 | 0.001 |
| N02AA05 | OXYCODONE | 1198 | 277 | 176.72 | 1.58 | 101.77 | 29.81 | 0.001 |
| C03CA01 | FUROSEMIDE | 2629 | 532 | 387.81 | 1.39 | 148.99 | 29.78 | 0.001 |
| R06AD02 | PROMETHAZINE | 483 | 136 | 71.25 | 1.92 | 65.14 | 28.76 | 0.001 |
| A15zzzz | APPETITE STIMULANTS | 213 | 75 | 31.42 | 2.40 | 43.69 | 27.47 | 0.001 |
| A06AB56 | SENNA GLYCOSIDES~COMBINATIONS | 396 | 104 | 58.41 | 1.79 | 45.81 | 17.73 | 0.001 |
| A03FA01 | METOCLOPRAMIDE | 229 | 66 | 33.78 | 1.96 | 32.31 | 14.86 | 0.001 |
| A06AA02 | DOCUSATE | 1250 | 255 | 184.39 | 1.39 | 71.71 | 14.70 | 0.001 |
| A03AX13 | SILICONES | 424 | 104 | 62.54 | 1.67 | 41.67 | 13.99 | 0.001 |
| A02AF02 | ORDINARY SALT COMBINATIONS AND ANTIFLATULENTS | 189 | 56 | 27.88 | 2.01 | 28.19 | 13.59 | 0.001 |
| A04AD10 | DRONABINOL | 48 | 22 | 7.08 | 3.11 | 14.93 | 13.16 | 0.001 |
| J01MA02 | CIPROFLOXACIN | 1521 | 296 | 224.36 | 1.33 | 72.99 | 12.65 | 0.001 |
| N02AB03 | FENTANYL | 121 | 39 | 17.85 | 2.19 | 21.18 | 11.69 | 0.001 |
| A12BA01 | POTASSIUM CHLORIDE | 1534 | 290 | 226.28 | 1.29 | 64.94 | 10.01 | 0.002 |
| A07AA02 | NYSTATIN | 205 | 53 | 30.24 | 1.76 | 22.82 | 8.56 | 0.006 |
| B01AB04 | DALTEPARIN | 25 | 12 | 3.69 | 3.26 | 8.31 | 7.74 | 0.018 |
| A02BC01 | OMEPRAZOLE | 2458 | 429 | 362.58 | 1.19 | 68.48 | 7.03 | 0.037 |
| V06DXzz | OTHER COMBINATIONS OF NUTRIENTS | 56 | 19 | 8.26 | 2.30 | 10.75 | 6.40 | 0.078 |
| N05AB04 | PROCHLORPERAZINE | 614 | 123 | 90.57 | 1.36 | 32.67 | 6.29 | 0.088 |
| A02BX02 | SUCRALFATE | 93 | 27 | 13.72 | 1.97 | 13.30 | 6.19 | 0.105 |
| A03AA07 | DICYCLOMINE | 157 | 40 | 23.16 | 1.73 | 16.87 | 6.14 | 0.114 |
| L01EX02 | SORAFENIB | 171 | 42 | 25.22 | 1.67 | 16.81 | 5.66 | 0.172 |
| V06DBzz | FAT/CARBOHYDRATES/PROTEINS/ MINERALS/VITAMINS~COMBINATIONS | 156 | 39 | 23.01 | 1.70 | 16.02 | 5.60 | 0.178 |
| A05AA02 | URSODIOL | 121 | 32 | 17.85 | 1.79 | 14.17 | 5.56 | 0.183 |

# **Table S10**. Drugs with p<0.3 in Days 1-133 among people with chronic liver disease in a sensitivity analysis increasing the minimum number of observed cases from 5 to 10.

| **Node** | **Drug Description** | **Total Node Cases** | **Observed Cases in Window** | **Expected Cases in Window** | **Observed**  **/Expected** | **Observed-Expected** | **Test Statistic** | ***P* Value** |
| --- | --- | --- | --- | --- | --- | --- | --- | --- |
| A06AD11 | LACTULOSE | 1951 | 1170 | 914.05 | 1.29 | 262.21 | 69.11 | 0.001 |
| C03DA01 | SPIRONOLACTONE | 2260 | 1319 | 1058.81 | 1.25 | 267.58 | 61.81 | 0.001 |
| C03CA01 | FUROSEMIDE | 2629 | 1486 | 1231.69 | 1.21 | 262.76 | 50.94 | 0.001 |
| A06AB06 | SENNA GLYCOSIDES | 792 | 505 | 371.05 | 1.37 | 135.26 | 46.16 | 0.001 |
| N02AA01 | MORPHINE | 652 | 418 | 305.46 | 1.37 | 113.44 | 39.54 | 0.001 |
| A15zzzz | APPETITE STIMULANTS | 213 | 154 | 99.79 | 1.55 | 54.35 | 28.44 | 0.001 |
| A04AA01 | ONDANSETRON | 1200 | 690 | 562.20 | 1.23 | 129.70 | 27.70 | 0.001 |
| N02AA05 | OXYCODONE | 1198 | 685 | 561.26 | 1.22 | 125.58 | 26.00 | 0.001 |
| A06AB56 | SENNA GLYCOSIDES~COMBINATIONS | 396 | 237 | 185.53 | 1.28 | 51.72 | 13.51 | 0.001 |
| L01EX02 | SORAFENIB | 171 | 113 | 80.11 | 1.41 | 32.96 | 12.84 | 0.001 |
| A03FA01 | METOCLOPRAMIDE | 229 | 145 | 107.29 | 1.35 | 37.82 | 12.56 | 0.001 |
| A06AA02 | DOCUSATE | 1250 | 671 | 585.63 | 1.15 | 86.70 | 11.86 | 0.001 |
| A12BA01 | POTASSIUM CHLORIDE | 1534 | 809 | 718.68 | 1.13 | 92.05 | 10.85 | 0.001 |
| N02AB03 | FENTANYL | 121 | 82 | 56.69 | 1.45 | 25.35 | 10.78 | 0.001 |
| A03AX13 | SILICONES | 424 | 246 | 198.64 | 1.24 | 47.60 | 10.66 | 0.001 |
| A02AF02 | ORDINARY SALT COMBINATIONS AND ANTIFLATULENTS | 189 | 120 | 88.55 | 1.36 | 31.53 | 10.58 | 0.001 |
| R06AD02 | PROMETHAZINE | 483 | 275 | 226.29 | 1.22 | 49.00 | 9.91 | 0.001 |
| A02BX02 | SUCRALFATE | 93 | 64 | 43.57 | 1.47 | 20.45 | 9.15 | 0.005 |
| J01MA02 | CIPROFLOXACIN | 1521 | 792 | 712.59 | 1.11 | 80.91 | 8.46 | 0.011 |
| P01AB01 | METRONIDAZOLE | 230 | 138 | 107.76 | 1.28 | 30.33 | 8.01 | 0.020 |
| N05AB04 | PROCHLORPERAZINE | 614 | 332 | 287.66 | 1.16 | 44.68 | 6.46 | 0.097 |
| V06DBzz | FAT/CARBOHYDRATES/PROTEINS/ MINERALS/VITAMINS~COMBINATIONS | 156 | 95 | 73.09 | 1.30 | 21.96 | 6.20 | 0.126 |
| A02BC01 | OMEPRAZOLE | 2458 | 1236 | 1151.58 | 1.08 | 87.04 | 5.99 | 0.138 |
| B05AA01 | ALBUMIN | 30 | 23 | 14.06 | 1.64 | 8.95 | 5.57 | 0.204 |
